# Determinants of relapse periodicity in *Plasmodium vivax *malaria

**DOI:** 10.1186/1475-2875-10-297

**Published:** 2011-10-11

**Authors:** Nicholas J White

**Affiliations:** 1 Mahidol Oxford Tropical Medicine Research Unit, Faculty of Tropical Medicine, Mahidol University, 420/6 Rajvithi Rd, Bangkok, 10400, Thailand

## Abstract

*Plasmodium vivax *is a major cause of febrile illness in endemic areas of Asia, Central and South America, and the horn of Africa. *Plasmodium vivax *infections are characterized by relapses of malaria arising from persistent liver stages of the parasite (hypnozoites) which can be prevented only by 8-aminoquinoline anti-malarials. Tropical *P. vivax *relapses at three week intervals if rapidly eliminated anti-malarials are given for treatment, whereas in temperate regions and parts of the sub-tropics *P. vivax *infections are characterized either by a long incubation or a long-latency period between illness and relapse - in both cases approximating 8-10 months. The epidemiology of the different relapse phenotypes has not been defined adequately despite obvious relevance to malaria control and elimination. The number of sporozoites inoculated by the anopheline mosquito is an important determinant of both the timing and the number of relapses. The intervals between relapses display a remarkable periodicity which has not been explained. Evidence is presented that the proportion of patients who have successive relapses is relatively constant and that the factor which activates hypnozoites and leads to regular interval relapse in vivax malaria is the systemic febrile illness itself. It is proposed that in endemic areas a large proportion of the population harbours latent hypnozoites which can be activated by a systemic illness such as vivax or falciparum malaria. This explains the high rates of vivax following falciparum malaria, the high proportion of heterologous genotypes in relapses, the higher rates of relapse in people living in endemic areas compared with artificial infection studies, and, by facilitating recombination between different genotypes, contributes to *P. vivax *genetic diversity particularly in low transmission settings. Long-latency *P. vivax *phenotypes may be more widespread and more prevalent than currently thought. These observations have important implications for the assessment of radical treatment efficacy and for malaria control and elimination.

## Introduction

In endemic areas of Asia, Oceania, Central and South America, and in the horn of Africa *Plasmodium vivax *malaria is a major cause of morbidity. It is an important contributor to early pregnancy loss and reduced birth weight which increases mortality in infancy [1,2]. *Plasmodium vivax *is a sophisticated and resilient malaria parasite which was once prevalent over much of the inhabited world. It has receded from North America, Europe and Russia, but in the tropics vivax malaria remains a major cause of childhood illness. In most endemic areas, *P. vivax *cohabits with *Plasmodium falciparum*. Mixed infections with the two species are common. *P. vivax *is more difficult to control and eliminate than *P. falciparum *because of its tendency to relapse after resolution of the primary infection. In endemic areas relapse of vivax malaria is a major cause of malaria in young children, and an important source of malaria transmission. Relapse also occurs in *Plasmodium ovale *infections and in several of the simian malarias, notably *Plasmodium cynomolgi*, which has often been used as an animal model of vivax malaria. The factors which control relapse and determine their remarkable periodicity are not known.

### History

**Figure 1:**
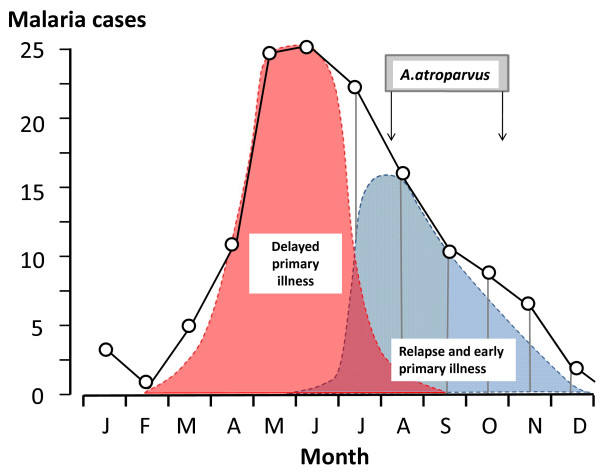
**Long-latency *P. vivax *in the Netherlands; The mean monthly number of malaria cases in the village of Wormerveer, The Netherlands, recorded by Korteweg from 1902 to 1923 (black line) **[15-17,19]. Swellengrebel et al showed that malaria transmission usually occurred between the first week of August and the end of October [16]. From this it can be deduced that the initial wave of malaria cases derived from inoculations the previous year (pink curve) and, by subtraction, that this was followed by relapses and primary cases with a short incubation period in the late summer and autumn (blue curve) [19].

**Figure 2:**
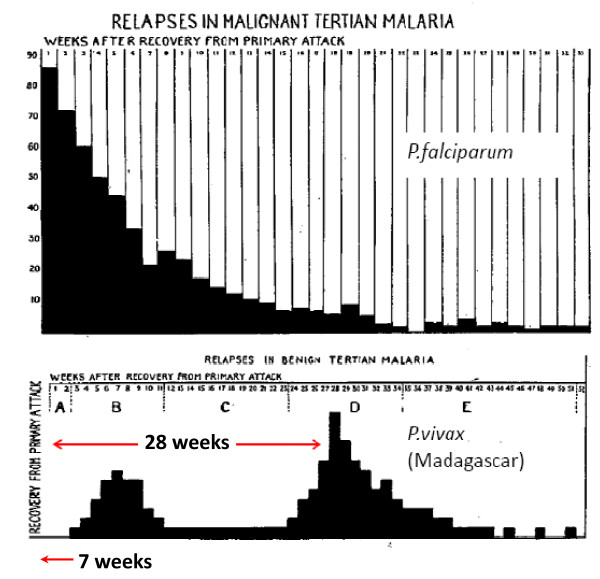
**The temporal pattern of illness recurrence in patients with neurosyphilis artificially infected for malaria therapy with *Plasmodium falciparum *(87 patients) and the "Madagascar" strain of *P. vivax *(105 patients) studied by SP James and colleagues at the Horton Hospital, Epsom, England **[24-26]**between 1925 and 1930**. The vivax relapses had a bimodal pattern with the majority having a long latent period (mode 28 weeks) before the relapse.

The history of clinical research on vivax malaria contains a wealth of valuable information that is not widely known or recognised. Most studies were conducted over fifty years ago and are not readily accessible to the modern reader. Indeed more may have been forgotten than has been learned about vivax malaria in recent years. The tendency of malaria infections to recur was well known since Roman times. From the 1630s onwards a specific treatment (Cinchona bark) was available for agues (although for most of those infected it was unaffordable). This treatment was often followed by frequent relapses of the periodic fever, and so opinions differed widely on its efficacy. Following Laveran's discovery of the blood parasite that caused malaria in 1880 [3], understanding of malaria epidemiology, pathology, and treatment was placed on a rational basis. In 1885, Golgi in Pavia distinguished the parasites responsible for tertian and quartan fevers [4]. In 1890, *P. vivax *was identified as a separate species by Grassi and Feletti [5], although debate continued into the early 1920s as to whether there were indeed separate human malaria species, or just one single polymorphic species [6]. Studies of the time often tended to consider the responses of the benign (vivax) and malignant (falciparum) tertian malarias together. The Dutch physician Pel is generally credited with the first postulation of an exoerythrocytic stage of malaria in 1886 [7]. In 1897, the American physician WS Thayer gave a very clear description of a long-latency relapse of malaria 21 months after the initial attack in a physician who had probably not been re-exposed between the two events [8]. Thayer and Bignami (in Italy) [9] both surmised that relapses of malaria resulted from a "spore" deposited in the internal viscera which remained inert "only to be set free as a result of some insult, the nature of which is still not appreciable to us" [8]. Self-experimentation then provided remarkably accurate descriptions of long latencies in vivax malaria. In 1900 in London Sir Patrick Manson was investigating the mosquito transmission theory proposed by his protégé Ronald Ross. Upon request Bignami and Bastianelli kindly provided him with *P. vivax *infected anopheline mosquitoes that had been fed on malaria patients in the Ospedale Spirito Santo in Rome. The mosquitoes were taken to the British Embassy in Rome, and thereafter by the "Indian mail", and duly arrived in London 48 hours later. His son Patrick Thorburn Manson, and George Warren (a laboratory technician) both volunteered to be bitten. Acute attacks of malaria followed after a two-week incubation period, in September 1900. These were treated successfully with quinine [10]. On June 1st the following year, after a latent period of nine months, the younger Manson experienced a sudden onset of rigors. Relapse of his vivax malaria was confirmed by microscopy [11]. Meanwhile in India, at the end of 1900, Major CF Fearnside infected himself with mosquitoes which had fed on vivax malaria patients in the Madras jail. His primary attack of malaria began on January 14th, he suffered a relapse on March 11 1901, and a second relapse followed on November 11th apparently without the possibility of interim reinfection [12]. These precise observations describing latencies of some eight to nine months were complemented by Korteweg's detailed and painstaking prospective epidemiological observations over more than two decades in the village of Wormeveer in The Netherlands. Celli had long surmised that the spring wave of benign tertian (*P. vivax*) malaria in Northern Italy resulted from relapse [13,14] but it was Korteweg who provided convincing evidence that the vivax malaria which emerged in the early summer had been acquired in the autumn of the previous year [15-19] (Figure 1). The considerable vivax malaria experience of the First World War (in the Balkans, Mesopotamia, and the Jordan valley), the secondary cases in England which followed the year after the return of infected soldiers, studies in American soldiers serving in Panama and the Philippines, studies in British soldiers serving in India, studies in Japanese soldiers who had invaded China, and further epidemiological observations and individual case reports in Europe and the United States all pointed to a disease which could relapse within two months of stopping quinine treatment, but also tended to relapse some eight to ten months later (Figure 2). Until the 1920s it was common to recommend an eight-week course of quinine treatment for malaria [20]. Despite this protracted treatment relapses were common, but whether these resulted from persistence of the blood stage infection (i.e. recrudescence), or derived from a latent tissue stage was unresolved.

### Early observations on relapse

**Figure 3:**
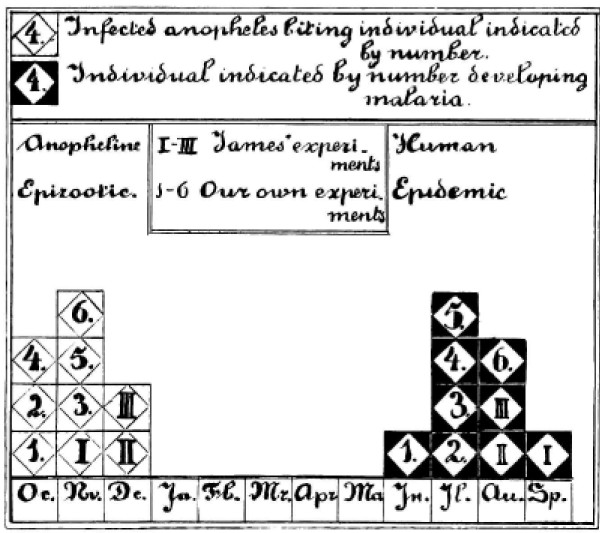
**Combined results of the preliminary mosquito infection studies of James and Shute in the Horton Hospital, Epsom, England and the self experimentation of Shüffner, Korteweg, Swellengrebel-de Graaf, Swellengrebel, de Buck and de Moor in The Netherlands as illustrated by Shüffner et al **[27]. This confirmed the 8 to 9 month interval from being bitten by one or two infected anopheline mosquitoes and developing vivax malaria

**Figure 4:**
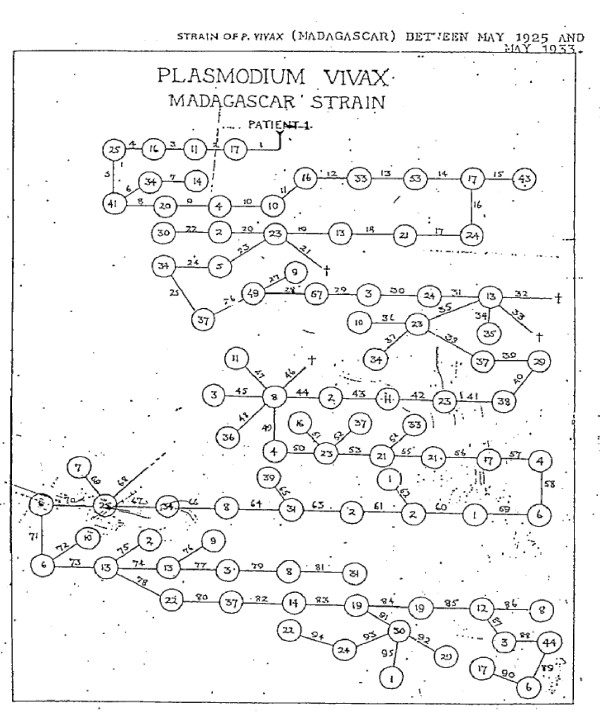
**The family history between May 1925 and May 1933 of the "Madagascar strain" of *Plasmodium vivax *as used in malaria therapy at the Horton hospital **[30]. The number within the circles refers to the number of patients infected with the "Madagascar strain" at each time and the number over the lines refers to the batch number of the infected mosquitoes. Overall 24,361 mosquitoes and 1739 patients were infected.

In 1913, Bignami proposed that relapses of malaria all derived from persistence of small numbers of parasites in the blood [21]. Although this theory explained the late recurrences of *Plasmodium malariae *infections in man, it did not explain several features of *P. vivax *and *P. ovale *infections. A much clearer understanding of relapse in *P. vivax *malaria was to come from Julius Wagner-Jauregg's discovery that malaria could cure neurosyphilis. Between the 1920s and the 1950s thousands of patients confined in mental hospitals with neurosyphilis were treated with malaria (malaria therapy). General paralysis of the insane was then a uniformly lethal condition, and at least half the malaria therapy treated patents were improved and over one fifth were cured. It was a remarkable period, unique in the history of medicine, when, as Henry Dale put it, "Man was the experimental animal". The majority of the malaria therapy experience was with a relatively small number of parasite "strains" transmitted either by blood passage, or more usually by the bites of several infected anopheline mosquitoes. On the 8th September 1922 Professor Warrington Yorke and JWS MacFie inoculated blood from a patient with simple tertian malaria (*P. vivax)*, which had been acquired in India, into a patient with neurosyphilis at the Whittingham Mental Hospital near Liverpool [22,23]. This "strain" was then used to infect multiple patients initially by blood passage, and later by mosquito transmitted infections. It soon became apparent that recurrences of *P. vivax *(and *P.ovale*) malaria followed a different temporal pattern to those of *P. falciparum *[24-26]. Recurrences of vivax malaria commonly occurred many months after apparently successful treatment of the primary infection. Furthermore whereas recurrence in blood transmitted vivax malaria could be prevented by curing the blood stage infection, recurrence in mosquito transmitted vivax malaria could not be [17,22-24]. This pointed to an exoerythrocytic stage of malaria, but its anatomical location remained elusive. The Dutch malariologists were able to show that their indigenous *P. vivax *could have a short incubation period if patients were bitten by a large number of infected mosquitoes, but, in a further example of courageous self-experimentation, they proved that bites by one or two infected mosquito were followed by vivax malaria 8 to 9 months later [26,27] (Figure 3). The Northern European and Russian "strains" of *P. vivax *therefore proved unsatisfactory for malaria therapy because blood passage was required to ensure an acute illness within weeks [17,20,22-29] whereas mosquito infection, even with multiple infected anophelines, often failed to produce an early febrile illness. Importantly it was also noted in The Netherlands that relapse rates in naturally acquired infections were higher than with mosquito-transmitted malaria therapy with local "strains" [17]. The most widely used parasite used for malaria therapy in Europe was a *P. vivax *"strain" isolated from an Indian merchant seaman whose last port of call had been Madagascar [8,21]. The "Madagascar" strain of *P. vivax *was extensively investigated in England by James and later in several European centres (Figure 2). It reliably produced an acute infection with high fevers, sometimes relapsed again within a few weeks, and then relapsed again approximately eight months later. With serial passage through man and mosquito the Madagascar strain apparently became even more virulent (Figure 4) [30].

**Figure 5:**
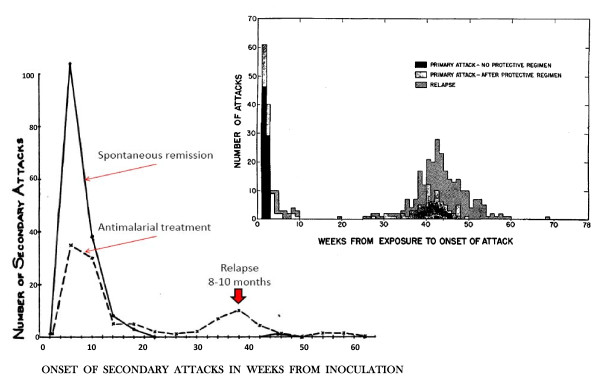
**Boyd's 10 year experience with mosquito transmitted *P. vivax *malaria therapy in 375 patients studied at the Florida State Hospital **[93]. Most infections were with the McCoy strain, and some were with other local "strains" which he considered to behave similarly. Infections which were allowed to continue until self termination did not relapse subsequently [31]. The median interval to relapse was approximately 9 months. Inset is the study of Coatney et al [71] describing 403 mosquito transmitted infections with the St Elizabeth strain of *P. vivax*.

James' terminology, which was adopted by several others, was different to that used today. A recurrence of malaria (any species) before eight weeks was termed a recrudescence, recurrence from 8-24 weeks was termed relapse, and return of malaria after 24 weeks was called a recurrence. The pattern observed by James in England, Swellengrebel in The Netherlands, and Ciuca in Romania with the Madagascar strain of *P. vivax *was very similar to patterns of the St Elizabeth and McCoy strains, of indigenous origin, used for malaria therapy in the United States [27-38]. These strains were all chosen because of their suitability for malaria therapy, so they probably represented the upper end of the spectrum of abilities to produce early infection and relapse. The European studies, investigations in India by Sinton and colleagues, and in Florida by Boyd suggested that some "strains" relapsed after a few weeks while others had only a primary infection, and then exhibited the long-latency seen with the Madagascar and St Elizabeth strains [24-30]. The distinction was often unclear as characterization of latency required reliable long-term follow-up, and the development of immunity with a febrile illness of many weeks duration became a significant confounder. As the object of malaria therapy was a prolonged high fever, the consequent long illness obscured early relapses. These single isolate (presumably single or highly related genotype) infections eventually resulted in solid immunity such that after several episodes of protracted fever reinfection with the same isolate was not possible [25-31,34,37]. This acquired immunity suppressed later homologous relapses. Importantly for our current understanding of *P. vivax *epidemiology asymptomatic parasitaemia (and gametocytaemia) tended to persist for weeks as disease controlling immunity was acquired. Boyd and Kitchen, who studied the McCoy strain in Florida extensively, noted that relapse did not follow infections which had terminated spontaneously (indicating an effective immune response) or infections in which the primary illness lasted for ≥ 48 days [31,34,37] (Figure 5). By contrast all agreed that if prompt anti-malarial treatment was given to terminate the infection then symptomatic relapses of vivax malaria were common.

During the malaria therapy experience it became clear that both the incubation period and the number of relapses were determined by the numbers of sporozoites inoculated [25-28,38]. The Dutch had shown first [27], and others later confirmed, that low sporozoite inocula often resulted in an extended incubation period of 7-10 months. The more sporozoites that were inoculated, the more likely was an early infection (incubation period two weeks), and the more relapses that followed - provided that prompt anti-malarial treatment (quinine) was given each time. Later clinical and experimental studies, reported after the Second World War, were to confirm these observations. In order to ensure that there was a short incubation period preceding the malaria illness malaria therapy infections were produced typically by the bites of 5-10 infected mosquitoes, and either no treatment or partial suppressive treatment was given. Previous theories that seasonal influences were important determinants of relapse were largely rejected as the intervals from primary illness to relapse in neurosyphilis patients were generally similar whichever month the infection started [22-26].

Between the 1920s to the 1940s the long-latency infection was regarded as the "usual" *P. vivax *phenotype. Throughout the endemic areas of Europe vivax malaria peaked in the late spring and early summer (largely from inoculations the previous year) [20,25]. In southern Europe there was often a bimodal pattern with a late summer peak of falciparum malaria [19]. The epidemiological studies of malaria epidemics in Sind (now Pakistan) and Ceylon (Sri Lanka) followed a similar pattern [19,25,39]. This suggested that long-latency *P. vivax *was also responsible for the peak of vivax malaria cases which occurred the year after the falciparum malaria epidemics in these two tropical areas (although other interpretations are also possible). During the Second World War observations on Allied soldiers fighting in North Africa, Italy, the Caucasus, and Greece, and further observations in Japanese occupation forces in China, all pointed clearly to long-latency *P. vivax *with similar illness patterns to the Madagascar and St Elizabeth strains [40-45]. In contrast the soldiers on both sides fighting in the Indo-Burman and South Pacific campaigns encountered vivax malaria with a very different relapse pattern. Relapses were frequent and the relapse rate was very high -in some companies all soldiers were infected and all relapsed. Infections occurred at three week intervals if quinine was given, and seven week intervals following mepacrine treatment [42-49]. Multiple relapses were very common and there was no evidence of long latency. The "type strain" for this tropical frequent relapse *P. vivax *phenotype was the "Chesson strain" isolated from a soldier of that name who had acquired the infection in New Guinea [50-52]. In volunteers infected with the Chesson strain 80% of relapses occurred within 30 days of initial treatment with quinine.

### Discovery of the liver stages

In 1902, three years before his death, the eminent protozoologist Fritz Schaudinn reported that he had observed direct infection of erythrocytes by sporozoites [53]. There was therefore no need to postulate a tissue stage of malaria. By the 1930s this theory was largely discredited as others had tried and failed to reproduce the observations, and by then tissue stages of bird malarias had been demonstrated unequivocally. The existence of a tissue stage of the malaria life cycle in humans was considered sufficiently likely that the Malaria Commission of the League of Nations in 1933 suggested that the sporozoite in human malaria went on to divide in cells of the endothelial system as did Haemoproteus in birds. In 1937 James and Tate showed that the exoerythrocytic development of *Plasmodium.gallinaceum *took place in the brain capillaries of chickens [54]. After the Second World War, Fairley's brilliant work at the Cairns experimental station showed that sporozoites were cleared from the blood within one hour of mosquito feeding on volunteers, and in the case of *P. vivax*, parasites only returned to the blood one week later [55-57]. Several researchers had noted earlier that in the latent or inter-relapse period even transfusion of as much as 500 mL blood could not transmit *P. vivax *to a volunteer recipient, whereas if *P. falciparum *recurred -it could always be transmitted beforehand by blood transfusion. It was clear then that there was a pre-erythrocytic tissue stage which preceded the blood stage infection, and also that subsequent relapses originated from an exoerythrocytic stage - but where was it? SP James, the eminent British malariologist, was so convinced that there must be an exoerythrocytic stage in the primate malarias that he told the young PCC Garnham to stay in East Africa until he had found it -and so he did! In 1947, Garnham identified the pre-erythrocytic development of *Hepatocystis (*then *Plasmodium) kochi *in the hepatocytes of African monkeys [58]. Shortly afterwards in England definitive studies by Shortt and Garnham identified the site of pre-erythrocytic development in primate malarias as the liver, first in *P.cynomolgi *infected Rhesus monkeys [59,60], and then in a heroic experiment with *P. vivax *in a very heavily infected volunteer who underwent open liver biopsy [61-63]. This classic work still did not identify the persistent stage, although later primate work suggested that relapses might arise from arrested development of hepatic pre-erythrocytic schizonts [63,64]. Forty years later Krotoski, working with Garnham and colleagues at Imperial College, finally identified the dormant stages or "hypnozoites" of *P.cynomolgi *and *P. vivax *responsible for relapses in the liver [65-69]. Although parasite bodies, which are probably hypnozoites, have since been demonstrated in liver cell cultures [70] remarkably little is known of their biology. The term relapse is now used specifically to describe recurrences of malaria derived from persistent liver stages of the parasite (hypnozoites) whereas recrudescence refers to a recurrence of malaria derived from persistence of the blood stage infection. The relapse arises after the "awakening" of these hypnozoites and the subsequent intrahepatic schizogony followed by blood stage multiplication. The question remained unanswered as to how the hypnozoites were woken, and what determined their remarkable periodicity.

### Phenotypic variation in *P. vivax*

**Figure 6:**
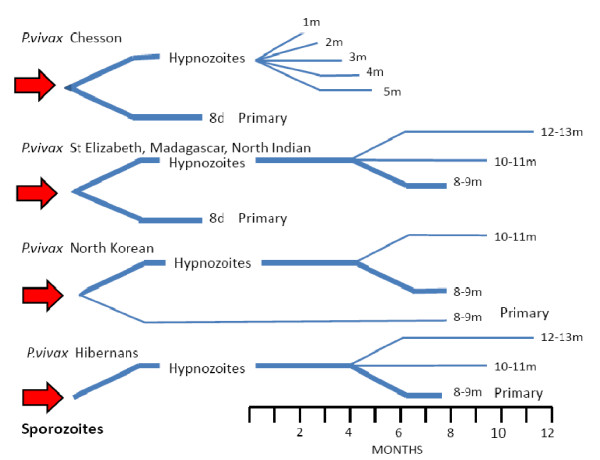
**Diagram of the different *P. vivax *phenotypes and the usual patterns of primary illness and relapse (after Bray and Garnham **[69]). The thickness of the lines gives a rough approximation of proportions.

Today there is a tendency to regard all *P. vivax *together as a single homogenous species, but the human malaria therapy and volunteer studies showed that there was substantial phenotypic variation between *Plasmodium vivax *"strains". There has been corresponding taxonomic debate over how these "sub-species" should be defined (Figure 6). Studies conducted over fifty years ago indicated that incubation periods, numbers of merozoites per blood schizont, antigenic relationships, intrinsic drug susceptibility, virulence, and relapse intervals all differed between "strains". At that time the *Plasmodium vivax *with infection phenotypes similar to those of the "Madagascar" and "St Elizabeth" strains which were prevalent in the United States and Southern Europe were considered the "typical" *P. vivax *infections [24,25,31,34,71]. A primary illness followed approximately two weeks after mosquito inoculation, and, although relapse could follow some three weeks later, there was often a 7 to 10 month interval before a subsequent relapse. Sometimes the latency could be as long as one year, and there were well documented, but apparently unusual, cases reported of latencies greater than two years. Further north in the Netherlands, Northern Germany, Scandinavia, Finland and Central Russia the long incubation phenotypes were prevalent (*P. vivax hibernans*) in which the primary infection occurred 8 to 10 months after inoculation [17-20,23,24,27-29,43,72-74]. It seemed that the proportion of infections which had a short incubation period (circa two weeks) declined steadily with increasing latitude (and shorter summer mosquito breeding seasons). In artificial infection studies latency was independent of season [22-26,71]. In the past twenty years infections with intermediate relapse characteristics have been reported but not well described from sub-tropical areas, although as will be explained later, there may be other explanations for relapses which emerge two to eight months after sporozoite inoculation.

**Figure 7:**
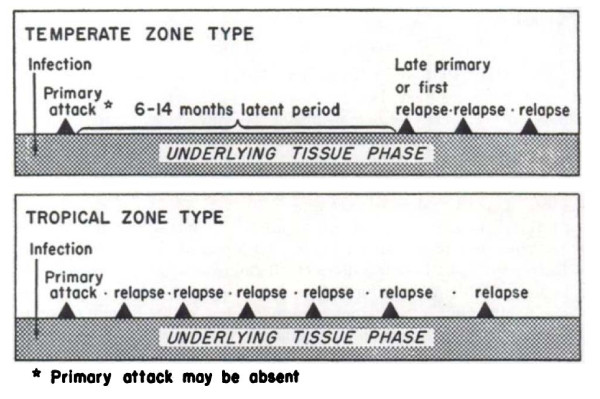
**Schematic diagram by Hankey et al **[79]**of relapse patterns following Korean vivax malaria (upper panel) and tropical frequent relapse *P. vivax *(lower panel)**. Note the frequent relapse pattern after a long interval with Korean vivax malaria.

There was one very important and puzzling feature of the long-latency *P. vivax *infections which still requires explanation in any theory which seeks to explain relapse periodicities; once the relapse had occurred (after a latency of 7-10 months) subsequent relapses would then usually occur with intervals of approximately 3 to 4 weeks following quinine -or 6 to 8 weeks if chloroquine was given for treatment [17,19,23,71-73] (Figure 7). Thus after the long latent period the subsequent intervals were similar to the incubation period in temperate infections with early relapses, and the inter-relapse intervals in the tropical frequent relapse "Chesson" phenotype vivax malaria [75]. In contrast infections from some parts of Russia were documented which had a second long-latency after the first relapse(s).

Following the Second World War artificial infection studies were conducted in several locations to study different anti-malarial treatment regimens. The administration of an effective schizontocidal drug allowed better definition of relapse periodicity than the malaria therapy studies (where the objective had been to produce a sustained high fever). In the more prevalent tropical strains of *P. vivax *treated in soldiers fighting in the Indo-Burmese and South-West Pacific campaigns in the Second World War, the relapses were documented to occur at intervals of 3 to 4 weeks (as in the Chesson strain) [42-52,76]. These observations were similar to those in the seminal chemotherapy studies of Sinton and colleagues in Kasauli, Himachal Pradesh, India in the 1920s and 1930s [32,77,78]. Interest in the long-latency phenotype revived during the early 1950s as *P. vivax *malaria which had a long-latency was an important problem in soldiers fighting in the Korean war [79]. During this period patients who had received very heavy inoculations, typically soldiers from the Second World War, would return to clinics for many years complaining of recurrences of malaria. The long latencies and the long relapse intervals in some infections and the multiple relapses in others despite anti-malarial treatment gave rise to the old saw that "you never got rid of malaria".

### Relapse intervals

#### Effects of sporozoite inoculum

**Figure 8:**
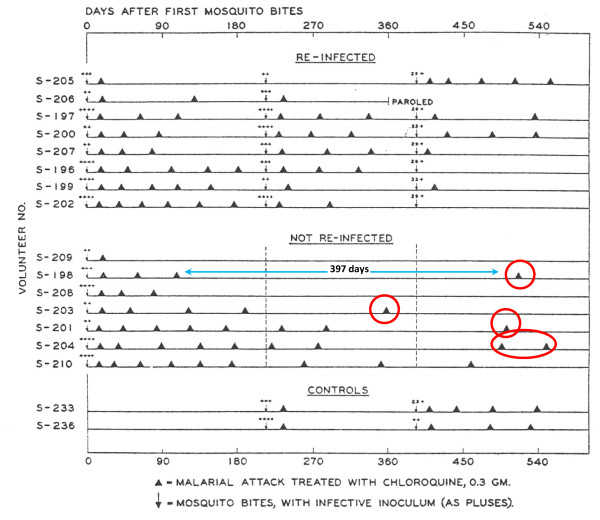
**Relapse patterns in volunteers in the USA who were infected by a single mosquito bite with the Chesson strain of *P. vivax *studied by Coatney et al **[75]. Some were rechallenged as indicated.

For long-latency vivax malaria from Northern climes the sporozoite dose determined the clinical phenotype [27-29,38,71-73]. In long term observations of infections with the St Elizabeth strain there was a clear bimodal pattern in which a long pre-patent period (~300 days) only occurred following smaller sporozoite inocula (more reflective of natural infection) [71]. Similar observations were made with a North Korean strain used for malaria therapy in a Moscow hospital from 1953 to 1968 [28]. When the inoculum of sporozoites was small (10-100 sporozoites) the initial parasitaemic illness did not occur for nine or 10 months, or longer. If ≥ 1000 sporozoites were inoculated illness occurred after a "normal" incubation period of two weeks. By contrast when increasing sporozoite doses of the tropical "Chesson" strain were inoculated the incubation period shortened and there was no evidence for a long prepatent period [75,80]. This, and a series of experimental investigations in chimpanzees [80,81], led Garnham to propose that the ratio of hypnozoites to immediately developing forms in the Korean *P. vivax *strain was 999:1 compared with the Chesson strain where he estimated the ratio as 50:50 [68,69]. (Figure 6) Several other important observations were made in the artificial infection studies conducted in humans and experimental primates. It was noted that even with a single infected mosquito bite some Chesson strain *P. vivax *infections relapsed after intervals which were as long as a year after a series of regular "short interval" relapses [75]. These terminal long intervals did not appear to have a fixed periodicity (Figure 8).

**Figure 9:**
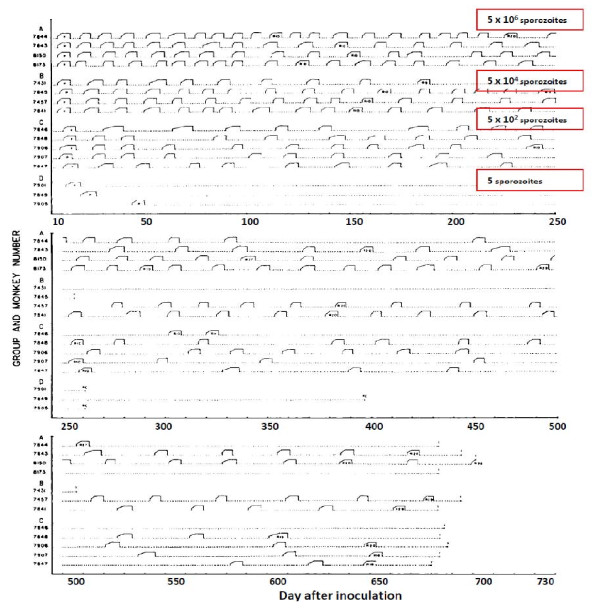
**Relapse patterns of *P.cynomolgi *infections in Rhesus monkeys studied by Schmidt **[82]. The infections were induced with different numbers of injected sporozoites as indicated and treated repetitively with chloroquine. Monkeys in groups A, B, C, and D were inoculated, respectively, with 5 × 10^6^, 5 × 10^4^, 5 × 10^2 ^and 5 sporozoites. Some monkeys were rechallenged and some were finally given radical treatment with primaquine in addition.

**Figure 10:**
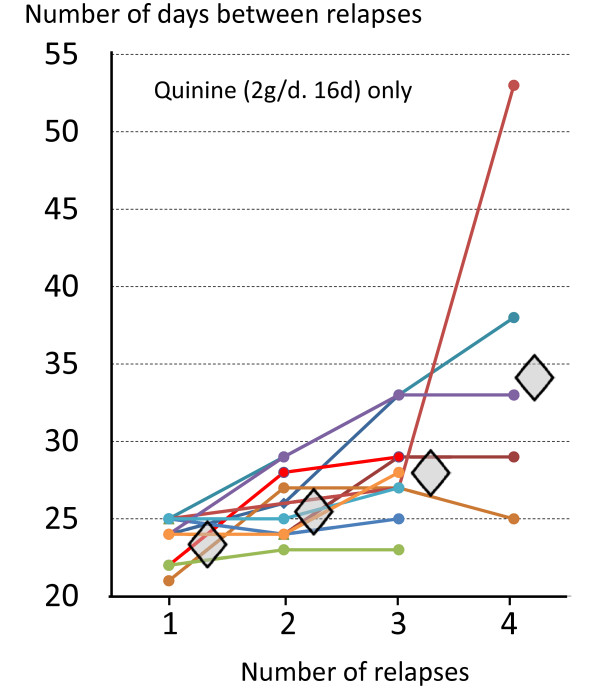
**Lengthening intervals between sequential relapses in individual volunteers who were infected with the Chesson strain of *P. vivax*, each of whom was treated with quinine for 16 days **[83]. Diamonds represent median values.

**Figure 11:**
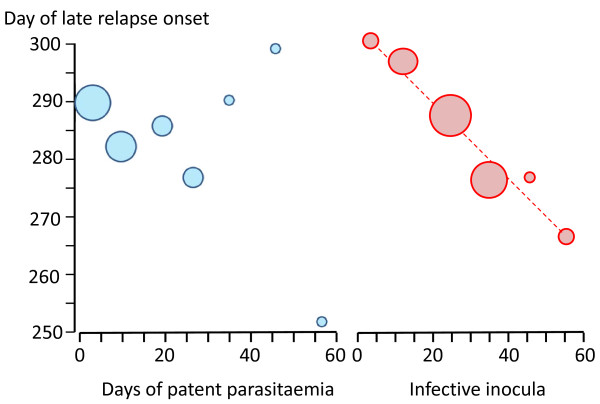
**In infections of volunteers with the St Elizabeth strain of *P. vivax ***[71], **there was no evident relationship between the occurrence or duration of the primary illness and the long-latency interval before illness (left panel), whereas there was an inverse relationship between sporozoite inocula (assessed semi-quantitatively from sporozoite numbers in the salivary glands and number of infectious bites) and the latency interval**.

In contrast the initial inter-relapse intervals of Chesson strain *P. vivax *in volunteers, and also *P.cynomolgi *in Rhesus monkeys, were remarkably regular although they gradually lengthened with each successive relapse [51,52,75,82,83] (Figures 9 and 10). Two factors are likely to have explained these gradually lengthening intervals - numerical probabilities and immunity. The simultaneous activation of several hypnozoites will shorten the relapse interval because the interval is measured until the progeny of the most rapidly growing parasites cause patent infection. For example if ten genetically identical hypnozoites are activated the relapse interval will in 91% of occasions be shorter than if one hypnozoite is activated. This is because there is natural phenotypic variation even amongst genetically identical organisms and it is the progeny of the earliest activated and most rapidly multiplying parasite that become patent first. This is best illustrated in the studies of Coatney et al with the long-latency St Elizabeth strain [71]. The interval from inoculation to relapse (~9 months later) shortened with increasing inocula. Thus the more hypnozoites that are activated the shorter is the average interval between relapses (Figure 11). This relationship is also clearly seen in Schmidt's studies of *P.cynomolgi *in monkeys [82] (Figure 9). This is an important consideration for concomitant activation of hypnozoites with different genotypes in endemic areas as will be discussed later. In natural settings multiple genotypically distinct hypnozoites may be activated but, on many occasions, only one or two genotypes will be detected subsequently at clinical relapse. The other hypnozoites' progeny may reach patency later, or asexual growth may be suppressed by fever, illness, immune response, and treatment such that they never reach patency.

#### Effects of immunity

The second factor accounting for lengthening inter-relapse intervals in artificial infections was the acquisition of blood stage immunity against the single infecting genotype. In the studies of the St Elizabeth strain there was clear evidence that late relapses were attenuated if there was an early infection, but this did not affect the interval to latency (Figure 11), whereas with increasing size of sporozoite inoculum there was a corresponding shortening of the interval. These observations suggest that inoculum size is more important than immunity in determining the duration of latency or the inter relapse interval - at least for the first relapses with genetically homologous parasites [71]. In Schmidt's primate studies (Figure 9) the lengthening of inter-relapse intervals was less prominent than in the human investigations [51,52,71,82,83] and increasing inter-relapse intervals were seen only after 12 or so relapses [82], although in these experiments the inocula were very large, and all animals received chloroquine treatment which was given early (on the second day of patency). Schmidt also observed in the Rhesus monkeys infected with *P.cynomolgi *[82], as Coatney had done earlier in volunteers infected with the Chesson strain of *P. vivax *[75], that occasionally a very long interval would follow a series of short intervals. Thus the steadily increasing intervals between the homologous strain relapses result from both the "running out of hypnozoites" with successive relapses, which results in reversion to the mean intervals associated with single hypnozoite activation, together with slower asexual growth rates resulting from the acquisition of asexual stage immunity. Blood stage immunity against homologous strains of *P. vivax*, which persists for many months, was a consistent observation in malaria therapy and challenge studies [34,84]. Boyd noted that if the initial infection was allowed to run its natural course, then relapse did not occur and reinfection with the homologous strain was not possible [34].

#### Effects of drugs

**Figure 12:**
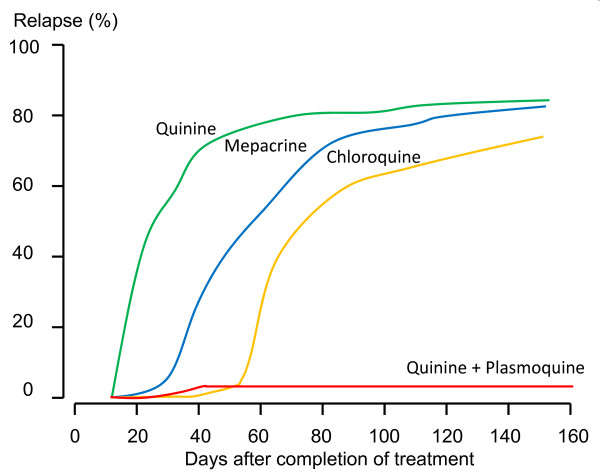
**Cumulative proportions of relapses in soldiers with vivax malaria acquired in the Pacific and treated subsequently with different anti-malarial drug regimens in Chicago**. Regimens were quinine; 2 g/day for 14 days (N = 75), mepacrine 0.4 g/day for 7 days (N = 69), chloroquine 1.5 - 2 g/day for 4-7 days (N = 82), and quinine 2 g/day and plasmoquine 60 mg/day for 14 days (N = 72) [47].

Since the introduction of mepacrine (atebrine, quinacrine) in 1932, a drug which has a terminal elimination half-life of over one month, it was observed that early relapses were delayed by approximately thirty days compared with quinine treatment (i.e. from three to seven weeks) [40,47]. Later chloroquine, which also has a terminal half-life of over one month, but a different elimination profile to mepacrine, was found to delay early relapse appearance by two to six weeks [40,47]. Larger doses of the anti-malarials resulted in longer intervals to relapse consistent with a concentration-dependent slowing of asexual growth rates. Slowly eliminated anti-malarials delayed the onset of *P. vivax *relapse, and consequently reduced their frequency, but importantly these drugs did not appear to reduce the overall number of relapses experienced [47] (Figure 12). Only the 8-aminoquinolines reduced or prevented relapses. The relapse preventing properties of these drugs, and their synergistic action with quinine, were demonstrated within years of their introduction in the mid 1920s [85-87].

Two other key observations were made during this early era of malaria therapy and drug evaluation which were not satisfactorily explained until decades later. It was noted that haemolytic reactions occurred sporadically with plasmoquine in patients of Asian, African or South European descent, but were uncommon in Caucasians originating further north [88]. This was explained later by the epidemiology of glucose-6-phosphate dehydrogenase deficiency. In the Southern United States it proved difficult of impossible to infect patents or volunteers of West African descent with *P. vivax *[34]. This was later shown to reflect the absence of the Duffy blood group receptor for *P. vivax *invasion of erythrocytes in this population.

### Contrasting artificial with natural infections

Artificial infections provided invaluable information but they differed from natural infections in several important respects [89]. The infections were in non-immune adults whereas the burden of vivax disease in endemic areas was in children. Adults in the malaria endemic areas have usually developed significant immunity to a broad range of local parasites which controls symptoms and reduces parasite densities. The artificial infections in the majority of volunteer studies and in malaria therapy followed the bites of 5-10 infected anopheline mosquitoes selected for maximal infectivity based on salivary gland sporozoite loads in sibling mosquitoes. The timing of inoculation in malaria therapy and experimental studies to coincide with maximum infectivity contrasts with the natural setting where anopheline mosquitoes display a wide range of infectivities depending on sporozoite age and other factors. The inocula in artificial infections were therefore usually "supranormal". This resulted in reliable infections but did not bring out the important stochastic component of *P. vivax *epidemiology resulting from low sporozoite inocula in areas of low seasonal transmission. Median sporozoite inocula in natural infections are estimated to be less than 10 sporozoites [90-92]. If Garnham's estimates (50:50 ratio of immediately developing parasites to hypnozoites) are correct this corresponds to a median of 5 hypnozoites in tropical *P. vivax *infections. The "strains" of *P. vivax *used in malaria therapy were likely to have been of a single (albeit evolving) genotype or very closely related interbreeding genotypes which were passaged through a very large number of patients over many years. Even if these infections originated as a mixed genotype infections in the donor patient it is likely that with multiple passage in malaria therapy the "strains" became purified through successive interbreeding to a very closely related group of genotypes. In contrast multiple unrelated genotype infections are common in natural infections.

The malaria therapy patients usually had neurosyphilis and were often very frail. Overall the mortality associated with *P. vivax* malaria therapy was approximately 7% (and was 10% with *P.malariae *infections -generally regarded as the mildest human malaria) [17]. This high mortality reflects the underlying condition, although it was undoubtedly contributed to by the infection as well. The objective of treatment in natural infections is cure, but in malaria therapy quinine was used to "damp down" the more severe infections, not to eliminate them. Reinfection with a different genotype was usual in endemic areas but in malaria therapy this was undertaken only occasionally if the first infection was insufficient, and in volunteer studies was performed to demonstrate the "strain specificity" of the immune response.

### The proportion of infections which relapse

The proportion of *P. vivax *infections which relapse is often thought of as an intrinsic property of the malaria parasites which varies considerably by geographic region. Tropical "strains" relapsed more than temperate "strains". But the relapse proportion is also clearly a function of the sporozoite inoculum and immunity (if any). As described earlier if artificial sporozoite induced infections were allowed to continue for weeks until self-termination the relapse did not usually occur and reinoculation was usually unsuccessful [34,84,93] (Figure 5). In this setting the probability of relapse with a presumed single genotype depended on the duration of preceding illness.

**Figure 13:**
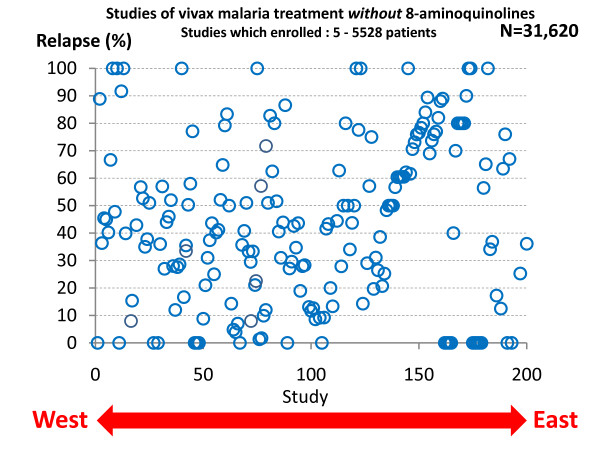
***P. vivax *relapse rates without radical treatment in published studies conducted between 1920 and 2010**. Each circle represents one study arm. The horizontal scale represents the study location from West (United States) to East (Pacific). A very wide range of treatments were used in a very diverse range of patient groups.

**Figure 14:**
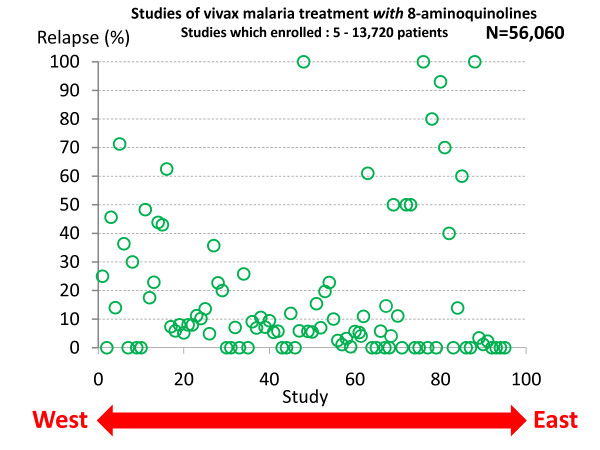
***P. vivax *relapse rates following treatments which included 8-aminoquinolines (mainly plasmoquine or primaquine)**. Otherwise as for Figure 13

**Figure 15:**
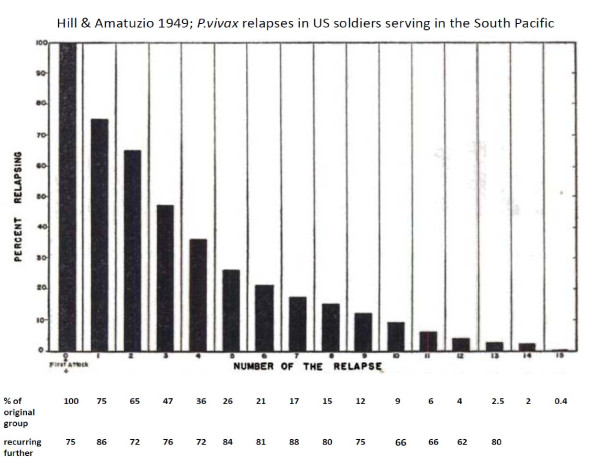
**Proportions of *P. vivax *relapses in 222 US servicemen who had fought in the South Pacific in the Second World War **[76].

In total, the treatment responses in over 87,000 patients with acute vivax malaria have been reported in the available medical literature in English (> 300 publications) of whom ~17,000 relapsed (Figure 13 and Figure 14). This experience of a wide variety of anti-malarial treatment regimens, is heavily biased to adults and military reports. Two-thirds of the reports are over fifty years old. The reported relapse rates varied from 0 to 100%! Many relapse rates are likely to have been underestimated as follow-up periods, particularly in recent studies, were often two months or less, and in the majority of studies radical treatments were being evaluated. On the other hand studies conducted in endemic areas could not exclude reinfection. The relapse rate was very high in soldiers who were generally non-immune and presumably experienced intense exposure. In the Second World War 70 to 80% of soldiers fighting in the South Pacific had relapses (and in many units all soldiers had relapses). In Hill and Amatuzio's series of 222 patients, 9% had 10 or more relapses [76] (Figure 15). Relapses were estimated to account for about half of all vivax malaria hospitalizations in the Second World War [94]. Once chemoprophylaxis was enforced in soldiers it became clear that almost all malaria could be prevented, but that several weeks after the mepacrine (quinacrine, atebrine) was stopped *P. vivax *relapses started to occur [47].

Without radical treatment the proportion of patients who experience one relapse, the proportion of these patients who have a second relapse, and the proportion of these who have a third and so on, is constant (Table 1). This appears to obtain in each different epidemiological or experimental setting [23,24,28,29,41,42,47-49,51,52,72,76,79,85]. In other words the relationship between the numbers of patients who experience one or more, two or more, three or more etc relapses is exponential.

**Table 1:** Relationship between the proportion of patients relapsing with vivax malaria and total number of relapses experienced

Proportion of incident *P. vivax *infections followed by ≥1 relapse (%)	Mean number of relapses per incident infection
**90**	8.3
**80**	4.0
**70**	2.3
**60**	1.5
**50**	1.0
**40**	0.67
**30**	0.43
**20**	0.25
**10**	0.11

### Thus if "x" is the fraction of patients experiencing one or more relapses, then the fraction experiencing "n" or more relapses is approximately x^n^

**Figure 16:**
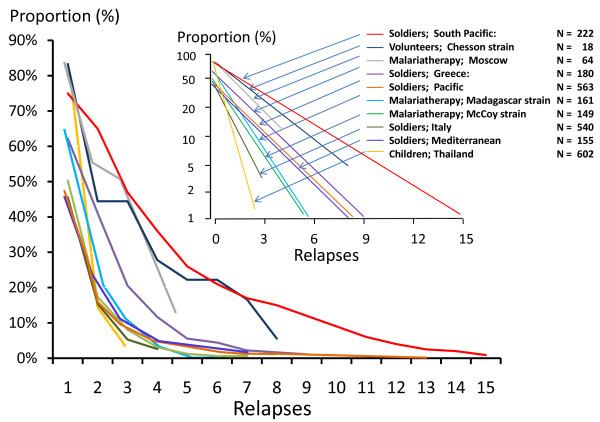
**The proportions of patients experiencing successive *P. vivax *relapses taken from eight different clinical series:** These were US soldiers with vivax malaria acquired in the South Pacific (2 series) [49,76], German soldiers who acquired vivax malaria in Greece [41], US soldiers with vivax malaria acquired in the Mediterranean area (two series)[45,47]and Italy[156], patients receiving malaria therapy with a local "strain" in Moscow [28], British patients receiving malaria therapy with the Madagascar strain [24,25], patients receiving malaria therapy with the McCoy strain in the United States [93], volunteers infected with the Chesson strain in the United States [75] and children followed prospectively in an evaluation of an ineffective malaria vaccine (SPf66) in northwestern Thailand [157]. Inset shows proportions on a log scale and numbers of patients studied.

A plot of the logarithm of the proportions versus number of relapses is, therefore, linear with slopes varying depending on the geographical and epidemiological setting (Figure 16). Thus, for areas with 50% relapse rates approximately 6.25% of patients have four or more relapses per incident symptomatic infection. This is important when considering radical curative drug efficacy as it provides an indication of the minimum burden of hypnozoites (because each relapse must derive from ≥ 1 hypnozoite) (text box on pharmacodynamics). It follows that once symptomatic relapse rates exceed 50%, then relapse becomes the predominant cause of vivax malaria illness.

### Geographic distribution

**Figure 17:**
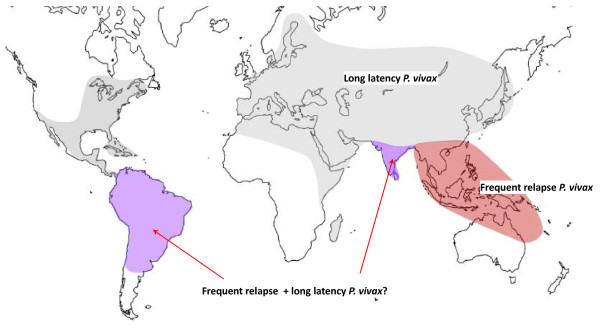
**Approximate historical distribution of *P. vivax *latency phenotypes**. Areas where tropical "frequent relapse phenotypes" are prevalent are shown in pink. Areas where both frequent relapse and long-latency phenotypes have been reported are shown in purple, and areas where long-latency phenotypes were prevalent are shown in grey. Although both South America and India are generally considered to harbour frequent relapse phenotypes predominantly, there is evidence that long-latency phenotypes are present in both areas (particularly across the North of India), and without genotyping it may be difficult or impossible to distinguish the two phenotypes within an endemic area (Figure 22).

Overall there was good evidence for the presence of the long-latency "Madagascar" relapse phenotypes in Europe, Central Asia, Southern Russia, North Africa, the horn of Africa, Madagascar, the Middle East, Afghanistan, Pakistan and Northern India, Central parts of China, Korea, North and Central America (Figure 17). Long incubation period *P. vivax *("*hibernans*") was prevalent in Northern Europe and more Northern parts of Russia. Frequent relapse "strains" were reported in parts of South America, India, South East Asia and Oceania. It is highly likely that in those areas where malaria has not been eradicated this geographic distribution of *P. vivax *phenotypes pertains today, although there is very little contemporary information apart from reports from South East Asia and the island of New Guinea (which has much higher levels of malaria transmission than most other *P. vivax *endemic areas of the world). It is also evident that both phenotypes overlap in geographic distributions (Figure 17). Where both are present together it would be very easy for the long-latency infections to go unrecognized, although the presence of a spring vivax malaria peak while vector densities are still low would be an epidemiological pointer [19,20]. There has been very little recent interest in this question despite its obvious importance. It is generally thought, but is by no means certain, that the majority of the global burden of vivax malaria today is the tropical frequent relapse type (although interestingly the first *P. vivax *to be sequenced fully was a long-latency phenotype from El Salvador-*Sal 1*) [95,96]. The greatest uncertainty is the epidemiology of relapse phenotypes in the Indian sub-continent, because India harbours the majority of *P. vivax *in the world and clearly has both phenotypes. This remarkable gap in our understanding of the global epidemiology of vivax malaria seems to have gone unrecognized outside the sub-continent.

Prospective studies from India over the past 25 years have recorded relapse rates following chloroquine treatment of between 8.6% (Orissa) and 8.9% (Madhya Pradesh) and 40.1% (Delhi) [97-99]. In Mumbai 19 out of 150 patients with vivax malaria and treated with chloroquine only and who were followed for one year had a relapse (17 within six months) [99]. Reinfection was considered unlikely. Higher rates were reported from the Delhi area [98] where the authors concluded "Based on the foregoing epidemiologic features, three distinct relapse patterns were observed in the present study, and it can be concluded that the *P vivax *population in northern India is polymorphic. Group I is the tropical or Chesson strain type of frequent relapsing *P. vivax *with a short period of latency between the primary attack and the first relapse, which is similar to other Southeast Asian strains such as those from Thailand and Vietnam. Group III is the temperate or St. Elizabeth-type strain that has a long period of latency between the primary attack and the first relapse. Group II is intermediate between these two types" [98]. This intermediate group, if it exists, has not been well characterized. The ratio of short to long-latency relapse phenotypes in Aligarh, Uttar Pradesh was estimated as 4:1 [100]. Overall relapse rates from India have been relatively low by comparison with South-East Asia [97-107]. Data from travellers returning from the Indian sub-continent have suggested that long incubation-period *P. vivax *may be common in the Punjab, and this is consistent with evidence in the past from Northern India, Pakistan and Afghanistan [108,109] and also the early observations of Fearnside, Yorke, and MacFie with artificial infections of Indian origin [12,22,23]. There are few data on temporal patterns of relapse in travellers in recent years, although studies in Eritrean immigrants to Israel, Turkish immigrants to Germany, and US soldiers returning from Somalia suggest the presence of long-latency phenotypes in the countries of origin [110-112]. Most travellers receive radical treatment for vivax malaria in temperate countries, which may be more effective against the long incubation or long-latency infections than against the tropical frequent relapse phenotypes.

The tropical frequent relapse phenotype was documented by Sinton and colleagues in British soldiers stationed in India in the 1920s and 1930s, and was observed in soldiers fighting in North-East India and Burma, and in prisoners of war in Thailand [46]. More recently Luxemburger et al studied 342 children with acute vivax malaria treated with chloroquine on the north-western border of Thailand in 1995 and 1996 [106]. Reappearance of *P. vivax *occurred in one patient on day 21 and in 8 by day 28, giving a 28-day cure rate of 97% [95% confidence interval (CI) 95-99%]. By day 63, the relapse/re-infection rate was 63% (95% CI 57-69%). Most reappearances of parasitaemia (85%; 121/143) were symptomatic. Silachamroon *et al *studied adult patients in Thailand (infections from Burma, Thailand, or Cambodia) with acute vivax malaria who were treated with either 5 days (N = 157) or 7 days (N = 159) of artesunate monotherapy [107]. Relapse rates within 28 days were 52.2% and 47.8% respectively. The timing of the relapses suggested that very few if any relapses emerged from the liver before the eighth day after starting anti-malarial treatment. In Papua Indonesia the relapse rate estimated at six weeks following artemether-lumefantrine treatment was 38% (the total number may well have been higher because of later emerging relapses) [113]. In French Guiana the relapse rate in children was 70% (nearly all relapses occurred within three months) but both recrudescence and reinfection could not be excluded [114]. Although South American *P. vivax *is generally regarded as "tropical frequent relapsing" in phenotype, a recent report from Rio de Janeiro that six of 80 travellers presenting with vivax malaria (who had returned from the Amazon region and not received chemoprophylaxis) had an incubation period of between three to 12 months, and another from Brasilia describing long-latency in three patients, suggest that long-latency forms may coexist with frequent relapse phenotypes in Brazil [115,116].

### The effects of age

**Figure 18:**
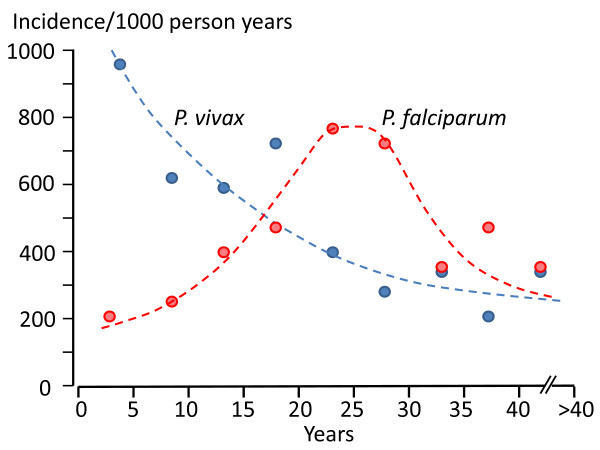
**The epidemiology of vivax and falciparum malaria in an area of low seasonal transmission on the Western border of Thailand; age incidence profiles **[106].

In malaria endemic areas, such as the north-western border of Thailand, the age profile of *P. vivax *malaria suggests much more rapid acquisition of immunity than for *P. falciparum *[117] (Figure 18). Entomological studies suggested similar transmission rates (at least in terms of measured entomological inoculation rates) so it is likely that relapse contributes to much of this difference. It also suggests that relapses are probably partially suppressed in older patients. Thus both the proportion of infections which relapse and the number of symptomatic relapses per mosquito sporozoite inoculation decline with age. It is likely that immunity and therefore age is a significant confounder in epidemiological assessments based on passive case reporting in many *P. vivax *endemic areas. In many areas adults are more likely to present to malaria clinics than children. In India the peak age of malaria presentation is often in young adults (and often with a predominance of young males). Yet in the indigenous population living in transmission areas a significant degree of immunity should have been gained (by both sexes) by early adulthood which reduces the number of relapses. Usually in endemic areas it is children who bear the brunt of malaria. This applies to both falciparum and vivax malaria when malaria is uncontrolled, but with increasing control falciparum declines more rapidly than vivax, and their epidemiology separates. The paucity of data from children may contribute to the low apparent relapse rates reported from the Indian sub-continent. It seems likely then that malaria clinic data are not necessarily representative of disease epidemiology in some endemic areas, and that studies of children living in the endemic areas of the Indian sub-continent might reveal higher relapse rates.

### Drug effects on relapse

Before the Second World War most malaria infections were treated with quinine. Early relapses of *P. vivax *were observed to occur approximately three to four weeks after starting quinine treatment. The 8-aminoquinoline plasmoquine (plasmochin, pamaquine) was evaluated clinically shortly after its discovery in 1924 in Germany. Sinton and colleagues in India soon provided evidence that plasmoquine synergized with quinine in the treatment of acute vivax malaria and also reduced the rate of recurrence (mainly relapse) [85,86]. "Sinton's regimen" of one week's quinine plus plasmoquine was endorsed by the League of Nations and generally replaced the two-month regimens previously in vogue [16]. In the 1930s the newly introduced sulphonamides were shown to have activity against malaria, but were more effective against *P. falciparum *than *P. vivax *[47]. The discovery of mepacrine (quinacrine, atebrine) in 1932 and its subsequent introduction provided a simpler, somewhat better tolerated, treatment although there was a pharmacokinetic interaction with plasmoquine which resulted in increased plasmoquine concentrations and oxidative toxicity, and precluded using the drugs simultaneously [118]. Mepacrine was very slowly eliminated and extensively distributed [119]. It was an effective treatment of vivax malaria. Mepacrine treatment markedly delayed the early relapse of tropical *P. vivax *which, usually then presented six to eight weeks after the primary infection rather than three weeks later, as was usual following quinine. When mepacrine was used as a prophylactic it suppressed infections for at least one month after stopping the drug [47,119,120]. Thereafter relapses followed soon afterwards in tropical areas where frequent relapse "strains" were prevalent, and many months later where long-latency "strains" were prevalent.

**Figure 19:**
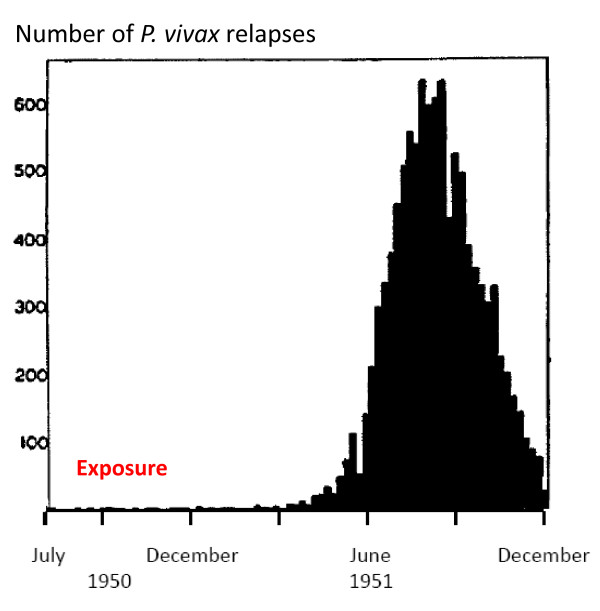
**Numbers of US servicemen admitted to hospital in the USA each week with vivax malaria following their return from the Korean war. Exposure was predominantly in 1950**. Figures taken from the Office of the US Surgeon General.

Extended follow-up studies suggested that although the relapses were delayed, *they were not prevented *[47] (Figure 12). It was assumed that the slowly eliminated treatment had suppressed the expansion of the blood stage infection (post treatment prophylaxis). It was unclear whether the relapse seen later was the first relapse which had been suppressed for three or more weeks, or whether the first relapse had been prevented altogether and it was in fact the second relapse (but the first clinical evidence of relapse) that became patent six to eight weeks after starting initial treatment. Mepacrine (atebrine, quinacrine) was the main drug used by all sides in the Second World War. The British were more willing to use plasmoquine than the US forces who discontinued its use during the war. They considered the risk exceeded the benefit [121], possibly because reactions were common in African-American soldiers (presumably G6PD deficient individuals who haemolysed). Relapse of vivax malaria was a major problem in soldiers, and was well documented in all the tropical theatres of war. Immediately after the Second World War the enormous research effort to find new anti-malarial drugs gave chloroquine (discovered in 1934 and initially overlooked), proguanil, and new more effective and less toxic 8-aminoquinolines. Chloroquine was clearly the best anti-malarial yet to be discovered, but like quinine and mepacrine, it acted only on the blood stage parasites. Proguanil, and later pyrimethamine, were both shown to have pre-erythrocytic activity but not radical curative activity. Furthermore resistance arose readily to the antifol anti-malarials in *P. vivax*. Although plasmoquine was moderately effective it was relatively poorly tolerated in the dose regimens necessary to prevent relapse (radical cure) [83-88]. The more effective and better tolerated 8-aminoquinolines pentaquine and then primaquine were developed and evaluated between 1944 and 1955 [122-126]. Primaquine took over as the standard radical treatment of vivax malaria (except in the USSR where the positional isomer quinocide was preferred). The 8-aminoquinolines also have significant blood stage activity against *P. vivax *(and *Plasmodium knowlesi*) although resistance in the blood stage infection can be induced experimentally [127]. The widely used chloroquine-primaquine regimen should therefore be considered a combination treatment. The 8-aminoquinoline development programme also uncovered the reason why some patients of African or Asian origin developed severe haemolysis, whereas Caucasian patients originating from Northern Europe did not haemolyse significantly. X-linked Glucose-6-phosphate dehydrogenase (G6PD) deficiency was discovered as the most common genetic defect of humans [128,129]. The dose of primaquine recommended globally was chosen largely as a result of studies on the relatively drug-sensitive Korean *P. vivax *[125,126,130]. After a very high rate of relapses was observed in US soldiers returning in 1950 from the Korean war (Figure 19), all soldiers were given a radical curative regimen of 15 mg base/day for two weeks during their return by sea [125]. This proved very effective. The Chesson strain had been shown to be more "resistant" to 8-aminoquinolines [124], but recommendations for a higher dose of primaquine (adult dose: 22.5 mg base/day) were applied initially only in Oceania. In hindsight there was no very good reason for this, and it might have been better to recommend higher doses for all frequent relapse parasites (i.e. in South-East Asia). It remains possible that all temperate "strains" are equally sensitive to primaquine, and all tropical strains are equivalent but have higher burdens of hypnozoites which could be activated immediately and so require a higher dose (i.e. 0.5 mg/base/kg for 14 days) (Table 2). This highlights the need for better discrimination of the tropical and temperate phenotypes and a better understanding of the epidemiology of relapse, and the pharmacokinetic-pharmacodynamic basis of radical treatment. One particular area of therapeutic relevance is the question of synergy. Sinton's work in India had suggested that quinine and plasmoquine were synergistic in the prevention of relapse in vivax malaria [85,86] and studies during and after the Second World War [47] provided further support for this notion. Ruhe *et al *showed that concurrent quinine and pamaquine (plasmoquine) was more effective in prevention of relapse with the St Elizabeth strain than sequential administration [131]. In the 1950s, Alving and colleagues conducted a formal interaction study which provided evidence of marked synergy between both quinine and chloroquine and primaquine [132]. The reason for this synergy (i.e. was it pharmacokinetic or pharmacodynamic?), and whether it extends to other quinolines or related compounds has not been explored further.

**Table 2:** Radical treatment pharmacodynamics; Quantitative considerations

Consider two groups of patients who represent two extremes	
Without radical treatment **group A **has an 80% relapse rate (eg some soldiers who fought in the Pacific in the Second World War)	
Without radical treatment **group B **has a 20% relapse rate (eg some soldiers who fought in the Korean War)	
Assuming a fixed fractional proportion of relapses and no acquisition of immunity, then the total number of relapses/100 patients is	
group A = 395,	group B = 24.
These numbers represent the ***minimum number ***of viable activatable hypnozoites (VAH) i.e. there are 16 times more in group A compared to group B. It is likely that the distribution of VAH is random among the patients and therefore conforms to a Poisson distribution.	
If primaquine at a dose of 0.25 mg base/kg (15 mg adult dose) reduces the number of viable activatable hypnozoites (VAH) by 90%, and there is no difference in susceptibility between the groups, and this effect is consistent across all patients then the post treatment number of VAH is	
group A = 39 or 40	group B = 2 or 3.
Thus we would expect 13 to 20 times more relapses in group A compared to group B.	
This hypothetical example simply points out that the apparent differences in primaquine "resistance" may reflect differences in the biology of the parasite rather than drug susceptibility per se.	

The mid 1950s saw a decline in clinical research on vivax malaria. Meanwhile most countries adopted the 15 mg base/day primaquine regimen usually given for 14 days. Five day regimens of primaquine (total adult dose 75 mg base) were recommended in the Indian subcontinent, although there was no evidence they were effective [97-105,133]. Baird has recently pointed out that the much improved tolerability of primaquine when taken with food was not emphasized sufficiently at this time and thus later dosage recommendations may have been limited by perceived or observed poor tolerability [134]. In the recent reawakening of interest in malaria it has been suggested that resistance to the radical curative activity of primaquine has emerged [135] - but it is not at all clear if there has been any significant change in susceptibility. As noted earlier, because the tropical phenotype is usually associated with a greater number of relapses than the temperate phenotype, then it requires a greater proportional reduction in activatable hypnozoites to prevent all relapses. More clinical pharmacology evidence on these important points is needed.

### Vivax malaria following falciparum malaria

**Figure 20:**
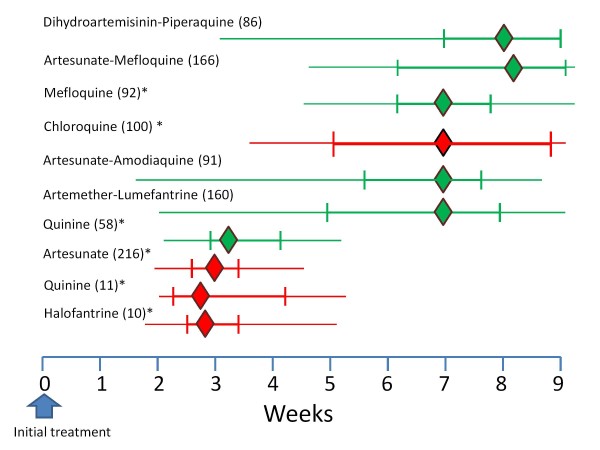
**The median (IQR, range) Intervals between acute *P. falciparum *malaria (in green) and acute *P. vivax *malaria (in red) and the subsequent vivax malaria episode in patients studied in Thailand following different anti-malarial treatments**. The figures in brackets are the number of patients studied [136-138]. * reinfection excluded.

In July, 1921 Major JA Sinton VC was put in charge of the Indian quinine and malaria inquiry under the newly formed Central Malaria Bureau. Between March 1924 and July 1925 Sinton compared two different quinine regimens in the treatment of falciparum malaria in British soldiers. These soldiers were followed for eight weeks at Kasauli (above the level of malaria transmission at an altitude of 6,000 feet in Himachal Pradesh). Of 76 soldiers completing follow-up 30 (39%) had subsequent vivax malaria [77]. In East Asia and Oceania a similar pattern pertains today. In East Asia a remarkably high proportion (20-50%) of symptomatic infections with *P. falciparum *are followed by an infection with *P. vivax *[136-138]. In Ethiopia where early relapse rates in vivax malaria are much lower, this proportion is also lower; 7% [139]. The intervals between the onset of treatment for the acute *P. falciparum *infection and the subsequent *P. vivax *infection are very similar to the intervals between acute vivax malaria and the first relapse (Figure 20). As the treatments given for falciparum malaria are highly effective, and therefore should be curative against the blood stage infections of *P. vivax*, these recurrent malaria infections are highly likely to be relapses. Furthermore, as with relapses after vivax malaria, the probability of vivax recurrence after falciparum malaria is also age related [138]. Several lines of evidence detailed below point to these vivax episodes being relapses of latent hypnozoites acquired *before *the *P. falciparum *inoculation.

### The periodicity of relapse

Various theories to explain the remarkable periodicity of *Plasmodium vivax *infections have been proposed [140] including reinfection of liver cells from released merozoites, intrinsic differences in latency periods of the inoculated sporozoites (tachyzoites, bradyzoites), and activation of dormant parasites by external stresses or seasonal stimuli [141,142]. In bird malarias there is reinfection of tissues from blood stage parasites. Following their seminal discovery of the pre-erythrocytic developmental stage in the liver, Shortt and Garnham initially suggested that this might also occur in the primate malarias [59-61]. However there is no convincing evidence to support this theory, and most are now agreed that reinfection from the blood stage back to the liver to produce secondary tissue stages does not take place in the primate malarias.

The temperate zone *P. vivax *usually had an incubation period of 8-9 months so emergence of an infection acquired in late summer or autumn could coincide with vector emergence in the following late spring or summer. Further south in temperate climes *P. vivax *infections had a primary illness two to three weeks after mosquito inoculation but the first relapse occurred 8-9 months later. Although the interval ("latency") between the primary infection and first relapse for this "Madagascar phenotype" was long (8-10 months), the subsequent inter-relapse intervals were short (Figure 7). In fact they were similar to the intervals from primary infection to early relapse which occurred in some Madagascar/St Elizabeth phenotypes and all Chesson phenotypes. This observation sits uncomfortably with a simple preprogrammed biological clock conjecture in which each sporozoite has a programmed latency. Lysenko *et al *pointed out that if the inoculated sporozoites were indeed a mixture of short and long-latency "zoites" then long latencies would only be evident if the sporozoite inocula were very small (otherwise there would almost invariably be one or more tachyzoites in the inoculum, and its emergence and subsequent treatment would obscure any later emergence of bradyzoites) [140]. Under this conjecture then perhaps failure to relapse early simply implies that long-latency (bradyzoites) only were inoculated. Much of the theorizing preceded discovery in 1982 of the dormant or persistent liver stage-now called the hypnozoite [65-69]. After the discovery of the hypnozoite the biological clock model was refined. The generally accepted theory (as described, and disputed, by Schmidt) has been that "sporozoite populations of all strains of *P. vivax, P. ovale*, and relapsing simian malarias such as *P. cynomolg*i, include a subpopulation that completes development promptly and is responsible for the early primary attack, and a group of subpopulations that undergo partial development to the resting hypnozoite stage. Subsets of these dormant pre-erythrocytic stages are *preprogrammed to resume development *at different times and, via this *built-in time clock*, evoke the sequence of relapses that characterize sporozoite-induced infections with the aforementioned plasmodia" [82]. As discussed previously it is difficult to understand how multiple relapses could occur at regular intervals with generally small inocula (median ~ five hypnozoites) and a simple clock mechanism. This remarkable efficiency suggests that some activation or feedback mechanism must operate in addition. A recent suggestion is that mosquito bites themselves might provide the trigger - perhaps by parasite sensing of a mosquito protein [143]. This is difficult to reconcile with the similarity of latency periods in indigenous peoples living in malaria endemic areas and the latency periods observed in malaria therapy patients and returned soldiers (i.e. away from seasonal mosquitoes and independent of season). There are relatively few recent data on relapse patterns in frequent relapse "tropical" vivax malaria, but the available evidence confirms the remarkable periodicity documented in the volunteer studies with the "Chesson strain". Most informative are studies in which anti-malarials such as quinine or the artemisinin derivatives have been used as these drugs are eliminated rapidly and do not suppress or delay the emergence of subsequent relapse [107,144,145]. Much of the early volunteer data with tropical "strains" was confounded partly by slowly eliminated drug effects and inoculations of unnaturally large numbers of sporozoites. Coatney realized this and so, in his classic series of 204 sporozoite-induced infections with the Chesson strain [75], he made detailed longitudinal observations following a single infected mosquito bite (Figure 8). The number of relapses varied considerably. In the seven volunteers who were not reinfected the median (range) number of relapses following a single bite was five (one to nine) and 11 of the 39 relapses in this group (28%) occurred more than six months after the initial infection. The interval from one relapse to the next was remarkably similar but overall the inter-relapse intervals gradually lengthened. Importantly there could then be very long intervals between relapses (four relapses occurred with preceding latencies exceeding six months. The maximum documented interval was 397 days). This proves that long-latency does occur with the tropical frequent relapse phenotype.

These patterns of relapse are also illustrated well in the primate model (Figure 9); the Rhesus monkey infected with *P.cynomolgi *(the primate malaria "equivalent" of *P. vivax*) [69]. In Schmidt's detailed longitudinal series [82] he noted that "The mean days separating each of the first four attacks (primary and first three relapses) were essentially identical in infections treated with chloroquine in combination with potentially curative agents, varying from 18.8 to 21.8 days from onset of patency in infections induced with the smaller inoculum and from 18.4 to 22.1 days in infections larger inoculums". In these monkey experiments where different sporozoite inocula were evaluated, it was noted that, although there was no clear difference in the number of relapses between monkeys inoculated with 5 × 10^2 ^sporozoites up to 5 × 10^6 ^sporozoites, "the intervals between relapses were related to size of inoculum, being distinctly shorter in monkeys inoculated with 5 × 10^6 ^sporozoites than in those challenged with 5 × 10^2 ^sporozoites, with recipients of 5 × 10^4 ^sporozoites occupying an intermediary position". Taken together with the absence of any relapses following an inoculum of only 5 sporozoites in three monkeys this argues for activation of a proportion of hypnozoites per relapse, and is consistent with the earlier observations in soldiers and malaria therapy patients of a fixed fraction of relapses following each illness episode in vivax malaria (Figure 16). It is unfortunate that inocula between 5 and 500 sporozoites were not studied in the primate model.

### The biology of relapse

Any theory seeking to explain the remarkable biology of *Plasmodium vivax *relapse must accommodate the following

1. Relapses show remarkable periodicity.

2. Early relapses reach patency around three weeks after starting treatment which suggests emergence from the liver at least one week earlier.

3. Not all *P. vivax *primary infections are followed by a relapse. In Thailand approximately 50% of infections are followed by a subsequent relapse within 28 days if a rapidly eliminated anti-malarial drug (artesunate) is given for treatment of the primary infection and primaquine is not given [107]. Elsewhere the probability of relapse generally varies between 20% and 80%. Animal experiments, the malaria therapy experience, and volunteer studies all suggest this proportion is a function of sporozoite inoculum.

4. Multiple relapses are common, particularly in young children, even though sporozoite inocula are thought to be relatively small (median 6-10 sporozoites). It is not uncommon in tropical areas for children to have four to six relapses at 4-6 week intervals and sometimes more following an incident infection. Even larger numbers of relapses were observed in soldiers following intense exposure and in Rhesus monkeys receiving very large sporozoite inocula. Importantly the fraction of people experiencing a relapse after each illness episode in a particular location appears constant

5. In long-latency phenotypes there is commonly a period of 8-9 months either before the first symptomatic infection, or between the first symptomatic infection and the first relapse. This long-latency interval appears to be normally distributed (mode 28 weeks for the Madagascar strain [24,25] (Figure 2). Sometimes there are several short interval relapses followed by a long interval. Conversely long latencies may also occur after multiple relapses in the tropical frequent relapse phenotype [75] (Figure 8).

6. if there are further relapses after the long latent period then they occur frequently with short intervals which are very similar to those observed in the tropical "strains".

7. The relapses in clinical studies conducted in endemic areas are commonly with a genotype which is different to that identified in the primary infection (48% in Columbian isolates, 55% in Indian isolates, 61% in Thai and Burmese isolates, and 71% in East Timor isolates) [146-148].

8. A remarkably high proportion of acute infections with *Plasmodium falciparum *are followed by an episode of *P. vivax *infection. The proportion is currently 30% in Thailand [136-138] and 50% in Myanmar [149]. The intervals between the acute *P. falciparum *malaria illness and the subsequent *P. vivax *malaria are similar to those between acute *P. vivax *malaria and the subsequent *P. vivax *relapse. The epidemiological characteristics suggest that these are all relapses (Figure 20).

It is also interesting and perhaps relevant that in endemic areas, despite often low seasonal transmission, *P. vivax *maintains a high degree of genetic diversity

### Relapses of vivax malaria arise from activation of latent hypnozoites (ALH)

Swellengrebel and de Buck [17] noted that relapse rates were higher in naturally acquired *P. vivax *infections (up to 50%) than in mosquito-borne infections with the local strains in neurosyphilis patients (despite larger inocula in the latter). They ascribed this to immunity, and this was undoubtedly a contributor, but another explanation is possible. That is that ***in endemic areas a high proportion of the population have latent P. vivax hypnozoites which can be activated by a sufficient stimulus***.

Four lines of evidence support this "Activation of latent hypnozoites" (ALH) hypothesis.

#### 1. Mixed species infections

Mixed blood stage infections with *P. falciparum *and *P. vivax *are underestimated by microscopy, but, even with sensitive PCR techniques, this proportion is insufficient to explain the 30% to 50% of patients in SE Asia who experience vivax malaria following falciparum malaria [138]. The interval between the primary *P. falciparum *infection and the subsequent *P. vivax *malaria strongly suggests this is a relapse (Figure 20). Persuasive supporting evidence that these are *P. vivax *relapses and not simultaneously acquired infections comes from entomology studies in which single anopheline mosquitoes have been examined for both species [150]. If these mixed species infections resulted from simultaneous inoculation then 30 to 50% of anopheline vectors carrying one species should also carry the other. In fact finding vectors with both *P. falciparum *and *P. vivax *sporozoites is relatively uncommon (overall in Asia 6.6% of *P. falciparum *sporozoite positive mosquitoes (17 of 258) also contained *P. vivax*). In the published literature not one of the 45 individually examined malaria positive wild anopheline vectors trapped in Thailand contained both malaria species [150]. This is also supported by the rarity of finding patients or healthy subjects with gametocytes of both species in the blood at the same time. Development rates in the mosquito are also slightly different (*P. vivax *being more rapid). Although space-time clustering of infections may occur in low transmission areas it is implausible that over 20% of *P. falciparum *inoculations would be associated with a separate *P. vivax *inoculation within one or two days, particularly when individuals receive on average less than one infectious bite per year. There is other supporting anecdotal evidence from travellers and from soldiers with brief periods of exposure in SE Asia. These groups have much lower rates of *P. vivax *following *P. falciparum*. The most plausible explanation for these findings is that the majority of these *P. vivax *episodes arise from hypnozoites which were latent in the liver of the patient at the time of acquiring *P. falciparum *(ALH). The remarkable similarity of both the timing of the *P. vivax *recurrences and their variance strongly suggest that latent *P. vivax *hypnozoites are activated by acute falciparum malaria.

#### 2. Heterologous genotypes

If *P. falciparum *malaria activates latent *P. vivax *hypnozoites then there is no reason why *P. vivax *malaria should not do the same. This would explain satisfactorily the finding of heterologous genotypes in one half to two thirds of *P. vivax *relapses [146-148]. In these cases where the original genotype was not detected in the malaria recurrence then either the inoculated infection did not relapse, or its hypnozoite(s) were activated but their progeny were outcompeted by the earlier activation or more rapid growth of the progeny of the activated latent hypnozoite(s). In approximately one third of *P. vivax *relapses studied in South East Asia, the malaria parasites isolated are either identical or closely genetically related to the primary infection. Relatedness could occur if there is little genetic diversity in the area where *P. vivax *malaria was acquired, or could result from recombination in the anopheline vector with the production of genetically related sibling sporozoites (i.e. simultaneous inoculation). If the "cross" took place several cycles of infection previously then subsequent infections may contain highly related parasites through successive interbreeding between related siblings. More work on this important area is needed. However in the majority of relapses the parasites are clearly genetically unrelated. In the one third of patients in whom the relapse is homologous or highly related with the primary infection, either there were no latent hypnozoites (as in volunteer studies), or the homologous infection's hypnozoites' progeny won the race to patency against the heterologous hypnozoites' progeny. It is evident then that close "races" between different genotypes to reach patency commonly result in gametocyte genotype mixtures in relapses (which may then recombine in the mosquito). Further support for the ALH hypothesis comes from observations in mothers and their infants living in a malaria endemic area on the north-west border of Thailand. The relapses of vivax malaria in the babies' mothers were usually genetically different to those which caused the primary infection, as in other patients studied in this area, whereas the relapses which followed the first *P. vivax *infection of life in their babies were usually of the *same *genotypes as those which caused the initial infection [151]. Obviously the infants could not have any previously acquired latent hypnozoites in their livers to be activated by the illness.

#### 3. Natural versus artifical infection relapse rates

Higher rates of vivax relapse in indigenous compared with artificial infection would also be explained by the ALH hypothesis. The incidence and number of relapses depends on the number of sporozoites inoculated. If all relapses derived from the inoculated infection then artificial infections which follow inoculations with 5-10 times more sporozoites should have higher, not lower rates of relapse. Relapse rates were particularly high in soldiers who were immunologically naïve and underwent intense exposure. If all relapses derived from the most recent inoculum then there should be no relationship between intensity of exposure and number of relapses.

#### 4. Long-latency also occurs in the tropical frequent relapse "Chesson" P. vivax phenotypes

Four of seven volunteers receiving a single infected mosquito bite in Coatney's series had relapses of the Chesson strain of *P. vivax *with variable but long latency periods - all exceeding six months after their preceding relapse [75]. It is not uncommon to encounter patients returning from malaria endemic areas where tropical phenotypes are prevalent with relapses more than 3 months after either a primary infection or return to the non-endemic area (of course these might also be long-latency phenotypes particularly if the interval is eight -10 months). This proves that some hypnozoites of frequent relapse phenotypes can remain dormant for long periods.

### Mechanisms of hypnozoite activation

If acute malaria activates latent hypnozoites and thereby causes vivax malaria relapses then it seems that a significant systemic illness is necessary for this activation. In the first half of the twentieth century, it was widely believed that a variety of external stresses could bring on a relapse of malaria [140]. By analogy, relapses in the bird haemosporidian parasite Leukocytozoon are provoked by the stresses of egg laying and the exhaustion of long migratory flights [152]. It was even taught in some textbooks of tropical medicine that Cinchona alkaloids should be given before surgery to pre-empt a relapse of malaria. However formal studies to provoke relapses of vivax malaria, which included forced route marches, simulated altitude, and induced hypoxia, did not yield convincing results [153,154]. Nevertheless it is interesting that soldiers in field hospitals in the Mediterranean region between 1940 and 1945 (who had acquired *P. vivax *in North Africa or Italy) were apparently considerably more likely to experience vivax malaria (presumably a relapse) if they had pneumonia or hepatitis compared with trench foot [155,156]. In our own series of children seen every day for over 18 months on the Thai-Burmese border during a study of the SPf66 malaria vaccine [157]*P. vivax *episodes were associated with malaria, but not with minor illnesses (S Lee; personal communication), so it seems that a substantial systemic stimulus is required.

**Figure 21:**
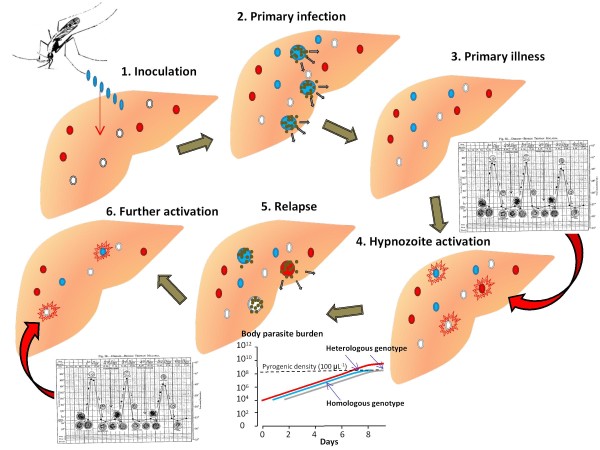
**Proposed mechanism and sequence of *Plasmodium vivax *relapse activation in a malaria endemic area**. In an illustrated example, at the time of infection (sporozoite inoculation) the individual already has hypnozoites of two different genotypes acquired from two previous inoculations which are latent in the liver. The different genotypes are denoted by different colours (red and white). Half the newly acquired infection sporozoites (blue) develop into preerythrocytic schizonts and half become dormant as hypnozoites (the estimated proportions for tropical "strains") [59,69]. Illness associated with the blood stage infection activates a small fraction of the hypnozoites (inset shows a "classic" *P. vivax *fever pattern in relation to asexual parasite development). In this example there is one hypnozoite of each genotype and each is activated by the illness and each develops into a pre-erythrocytic schizonts. By chance the progeny of one of the preexisting latent hypnozoites reach pyrogenic densities before the progeny of the recently inoculated hypnozoite (inset shows the logarithmic growth of the three genotypically different infections-vertical axis shows number of parasites in the body). The consequent febrile illness then suppresses further multiplication of the blood stage infection so that the progeny of the other two prerythrocytic schizonts may not reach transmissible densities. The ensuing illness activates some of the remaining hypnozoites (the same fraction as were activated initially) and relapses continue until either the number of hypnozoites is exhausted or some fail to be activated. If there are some hypnozoites which fail to be activated these may be activated at a later date by a subsequent malaria infection.

Periodicity can be generated in several ways in biological systems. Illness generally (and the associated cytokine responses) or malaria specifically could provide the activating or inhibitory necessary stimuli to generate periodic relapses in vivax malaria. Overall it seems most likely that **the illness associated with the infection itself **is the activator in *P. vivax *relapses (Figure 21), and that each symptomatic episode provides an activation stimulus which may give rise to the next relapse. A simple clock mechanism with a single unimodal distribution of latencies alone is inadequate to explain the observed phenomena. Equally, as explained previously, a multiplicity of different latencies with a single harmonic is very complex and difficult to reconcile with the efficient use of relatively small sporozoite inocula (median ~10 sporozoites). For efficient yet periodic activation of small numbers of hypnozoites (i.e. so that they do not all activate together) it is necessary either to hypothesise a negative feedback mechanism, which may occur with illness being the negative feedback, or propose simply a relatively broad temporal distribution of latencies (analogous to seeds germinating or eggs hatching), and a *low individual susceptibility *to activation. Either results in several relapses at regular intervals, each activated by the preceding illness. Importantly the proportion of susceptible hypnozoites activated would be fractional, at least initially, which fits with the observed fixed proportions of patients who experience multiple relapses (Figure 16). It also may leave a significant proportion of unactivated (but activatable) hypnozoites which remain latent until either they die (for example when the host hepatocyte dies) or they are activated by a systemic illness such as malaria. This would explain the high rate of vivax malaria (presumably relapses) following falciparum malaria. It follows that the number of immediate relapses per mosquito inoculation will decrease with increasing age in endemic areas, as the stimulus to hypnozoite activation declines with increasing disease controlling immunity, and immunity increases the probability that the relapse is asymptomatic. In this respect it is interesting that in Thailand the incidence of symptomatic *P. vivax *peaks in childhood [106], but *P. vivax *malaria following *P. falciparum *malaria shows a weaker age relationship. *P. falciparum *may contribute significantly to *P.vivax *transmission, particularly in young adults, through this mechanism. It also follows that with small inocula it is quite possible for all sporozoites to develop immediately (early infection, no relapses) or for all to result in hypnozoites, and all the hypnozoites to become latent (no early infection, first infection follows activation stimulus such as a malaria illness).

For temperate strains of *P. vivax*, as suggested by several investigators previously, there are clearly at least two populations of hypnozoites, one becoming activatable early (as for tropical *P. vivax*) and another remaining latent and not immediately activatable. The study of Cooper et al, in which blood induced homologous (St Elizabeth strain) infections were induced 120 days after infectious mosquito bites, is informative in this respect [35]. The blood inoculations reliably gave rise to symptomatic malaria but critically *did not *affect the timing of the subsequent relapse. This suggests that the hypnozoites (half way through their sleep) were absolutely refractory at this time. It is likely that once hypnozoites do become activatable, that there is a background relatively low rate of spontaneous activation in order to account for the distribution of latencies. Thus for the long-latency *P. vivax *the first relapse after the long latent period is usually spontaneous, but the illness then activates further hypnozoites, accounting for the subsequent short inter-relapse intervals. If this is correct it follows that *first *relapses which occur with a long-latency interval (i.e. 8-10 months after the primary infection) should usually be of a *similar *genotype to the initial infection, whereas relapses at other times could be heterologous. If there are subsequent relapses i.e. which follow the first relapse after the latent period (i.e. with a short periodicity) then these could be genetically heterologous.

*Plasmodium vivax *in temperate zones has clearly evolved to adapt to the long winters across the Northern hemisphere [17,19,27-29,79,158]. It is interesting to speculate that the large proportion of sporozoites dedicated to latency, and the relatively low number of relapses compared with the tropical strains may reflect the high natural wastage of hypnozoites in hepatocytes which die before the hypnozoites become activatable. In experimental *P. cynomolgi bastianelli *infection in the Rhesus monkey Garnham observed a ten-fold reduction in the number of hypnozoites in serial liver biopsies over a nine month period, but it is not clear how much of this reduction resulted from activation and how much was from cell death [68].

The hypnozoite can be considered as an unactivated sporozoite. If the duration of pre-erythrocytic development of the liver stage is similar for sporozoites and hypnozoites, as seems likely, this puts activation at the time or shortly after presentation with acute malaria (of any species). If the ALH hypothesis is correct then the key biological difference between frequent relapse and long-latency *P. vivax *phenotypes lies in the temporal distribution of susceptibility to activation among the sporozoites. This can then be subdivided into the proportion of sporozoites which activate immediately and the subsequent temporal distribution of susceptibility to activation among the remaining hypnozoites. The genetic basis for this regulation may be difficult to find, as it could well reflect subtle quantitative rather qualitative differences. Recent studies in murine malaria indicate a central role for iron sequestration in controlling pre-erythrocytic development through malaria illness induction of the iron regulatory hormone hepcidin [159,160]. It is possible that iron availability also plays an important regulatory role in controlling hypnozoite activation and pre-erythrocytic development. One possible mechanism contributing to regular short interval (three weeks) relapses is a malaria illness related temporary halt to liver-stage development (mediated by reduced iron availability), which is then lifted with clinical recovery. Some of these hypotheses may be testable in the ex-vivo hepatocyte culture systems [70].

In summary this ALH hypothesis proposes that there is indeed a biological clock (which is most evident in temperate strains) determining latency in *Plasmodium vivax*. This clock (which could be an intrinsic parasite clock, or could reflect a host-parasite interaction) determines the length of the interval before the hypnozoite becomes susceptible to activation, but that there is a separate sensing mechanism which determines whether or not activation does occur. The trigger could be either a positive activation stimulus or removal of inhibition. This mechanism may activate spontaneously once the hypnozoite has become susceptible, and spontaneous activation presumably usually explains the first relapse after a long latent period, but once susceptible activation is much more likely with an external systemic illness such as malaria. Activation must involve signaling via the infected hepatocyte (which is very sensitive to systemic inflammatory responses). Importantly the individual probability of activation for each hypnozoite is low, allowing accumulation of latent but "activatable" hypnozoites after each sporozoite inoculum. This implies that people living in vivax endemic areas commonly harbour latent but "activatable" hypnozoites. In endemic areas of South-East Asia, if patients who acquire falciparum malaria are representative of the population, this figure is approximately one quarter to one third. The periodicity of *P. vivax *relapses is derived from the sequential activation of hypnozoites by illness. Mathematical modeling will provide valuable insights into which activation-inhibition model best fits the observed malaria therapy, volunteer, and epidemiological information.

### Implications for epidemiological assessment

**Figure 22:**
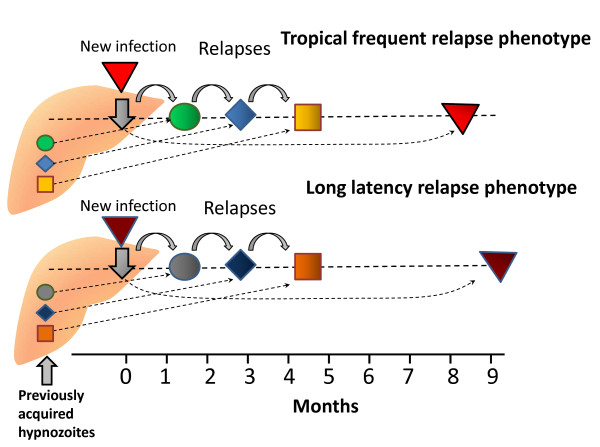
**Potential similarity of relapse patterns with the long-latency and frequent relapse *P. vivax *phenotypes**. The different colours and symbols represent different genotypes in two hypothetical infections. The upper panel shows the relapse pattern where the frequent relapse phenotypes are prevalent. The patient has hypnozoites from three preceding inoculations present at the time of illness from the newly acquired (red) infection. These are sequentially activated as proposed in Figure 21. Three relapses occur with similar periodicities, and a later randomly activated relapse occurs 8 months later. In the lower panel the long-latency phenotypes are present. Activation occurs as above and the long-latency relapse emerges nine months later. The initial relapse patterns associated with the two different phenotypes are identical. This illustrates the difficulty in excluding the presence of long-latency phenotypes in malaria endemic areas.

If the ALH theory is correct it also explains why relapse phenotypes in tropical malaria endemic areas may be difficult to characterize, and why in areas with long-latency *P. vivax *frequent relapse patterns may still be observed (Figure 22). This is because a primary illness with long-latency *P. vivax *of the "Madagascar" or "St Elizabeth" phenotype may activate previously acquired latent hypnozoites (of similar phenotype) -giving an early relapse. The illness caused by the relapse could then activate further latent homologous or heterologous hypnozoites. Thus several relapses may follow at short intervals even though all the parasites are of the long-latency type. The net result would be indistinguishable from the epidemiological pattern of frequent relapse vivax malaria (Figure 22). The converse -long-latency with the tropical frequent relapse phenotypes was demonstrated clearly in the studies of Robert Coatney and his colleagues [75]. If both types are prevalent in the same area then identifying phenotypes from illness patterns becomes even more difficult, and it may be impossible to discern the long-latency phenotypes amongst the frequent relapses. In temperate areas, such as Southern Europe, the early spring-summer wave of vivax malaria which preceded the appearance of anopheline vectors was an obvious clue, but in tropical regions with longer transmission seasons this may be difficult to discern [19]. The presence of long-latency phenotypes may only be evident in travellers and soldiers who spend a brief period in the endemic area, do not receive primaquine, and then return to a non-endemic area and relapse many months later without re-exposure [108-112]. These cases provide an important epidemiological signal. There is uncertainty over the true epidemiology of relapse patterns over a large proportion of the *P. vivax *endemic world. Despite a resurgence of interest in malaria, and the belated recognition of the importance of *Plasmodium vivax*, this large knowledge gap seems to have gone unnoticed. It is quite possible that long-latency phenotypes are present in many tropical areas (Figure 17). Defining these patterns is an essential prerequisite for therapeutic assessments, control and elimination planning, and the evaluation of novel interventions such as vaccines.

### Implications for the emergence of anti-malarial drug resistance

**Figure 23:**
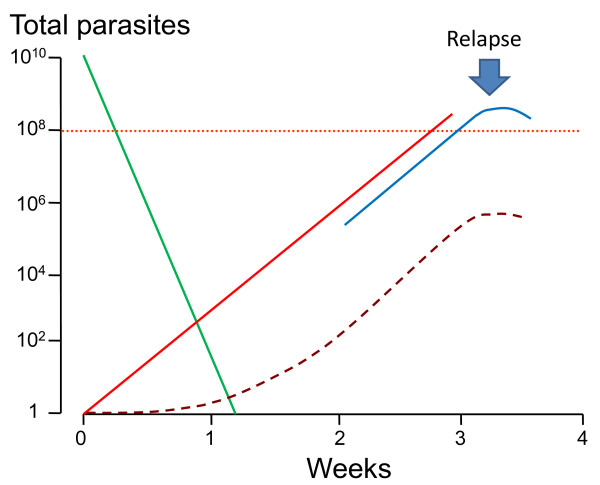
**Relapse pre-empts the emergence of de-novo anti-malarial drug resistance**. De-novo drug resistance is a rare event and usually there is only one mutant resistant parasite which multiplies while the sibling drug sensitive parasite population declines (green) [160]. Only a highly resistant parasites' progeny (red line) can reach transmissible densities in blood (total numbers circa 100,000,000 in the body) before the relapse (comprising drug sensitive parasites) becomes patent and the consequent illness (and any treatment effect) suppresses multiplication. Slowly eliminated anti-malarial drugs reduce this protective effect by reducing multiplication of the relapse parasites more than multiplication of the de-novo resistant parasites.

The factors determining de-novo selection of anti-malarial drug resistance are likely to be similar across all malaria parasites (pattern of drug exposure, parasite biomass, frequency and stability of the genetic or epigenetic mechanism, fitness cost) [161]. The much lower parasite biomass in *P. vivax *malaria makes de-novo selection less likely than in *P. falciparum *infections, some of which may comprise very large numbers of parasites (> 10^12^) [162]. In malaria, recrudescence is necessary for onward transmission of a de-novo resistant mutant parasites' progeny [161]. In *P. vivax *malaria the de-novo recrudescent infection is effectively in a race with the relapse-either of heterologous or drug sensitive homologous (sibling) parasites. Resistant parasites will only be transmitted if their progeny reach transmissible parasite densities *before *the relapse does. If the de-novo resistance is high grade then the resistant parasites may expand in numbers rapidly and may not be impeded by the relapse. However if the first step in resistance is a small reduction in susceptibility, as is more usual in anti-malarial drug resistance, then resistance cannot be transmitted onward in infections in which the relapse becomes patent before the recrudescence would have (Figure 23). This is because parasite multiplication falls rapidly once symptoms have developed, and treatment usually follows soon afterwards. Of course the resistant parasites are less drug-sensitive so they will have a "second chance" to recrudesce later if the same drug is used for treatment of relapse, but treatment may change, primaquine may be added, and immune responses are strengthening -all of which reduce the chance of subsequent recrudescence. The relapse therefore pre-empts the expanding intra-host population of de-novo resistant parasites. Together with the lower biomass, and the use of primaquine in "combination therapies", this may explain the slower emergence of low-grade anti-malarial drug resistance in *P. vivax *compared with *P. falciparum*.

Slowly eliminated drugs interefere with the resistance protection provided by relapse. Chloroquine has a slow multiphasic elimination phase and suppresses the relapse of sensitive parasites for approximately two weeks (from two to six weeks). Mefloquine has a different profile of elimination, and provides longer suppression of vivax relapse. The residual drug levels *reduce *potential interference with resistance emergence by relapse providing greater suppression of the sensitive compared with the resistant parasites and they therefore reduce the delay on resistance emergence. In summary early relapse provides a brake on the emergence and spread of low-grade resistance in *Plasmodium vivax *by pre-empting recrudescence.

### Implications for genetic diversity

Activation of hypnozoites from different preceding inoculations will commonly result in two or more genotypes reaching patent parasitaemia at similar times. As gametocytogenesis in *P. vivax *occurs simultaneously with asexual stage development this provides an effective method of increasing the likelihood that a mosquito will ingest gametocytes of different genotypes, thereby facilitating meiotic recombination between genetically unrelated *P. vivax *parasites. This must be an important contributor to the relatively high degree of genetic diversity in *P. vivax *often found in areas with very low seasonal transmission.

### Practical implications

If the ALH theory is correct then the epidemiology of malaria relapse and the biology of interaction between the species need reconsideration. If long-latency *Plasmodium vivax *still contributes a significant proportion of vivax malaria in the Indian sub-continent and further west then current methods of assessing drugs and vaccines may also need reconsideration (Table 3).

**Table 3:** Epidemiological implications

1.	A substantial proportion of the population in *P. vivax *endemic areas harbours latent but activatable hypnozoites. Some of these may derive from inoculations which were not followed by any illness.
2.	If relapse rates exceed 50%, then relapse becomes the predominant cause of vivax malaria.
3.	Spontaneous or activated relapse followed by asymptomatic parasitaemia may be an important source of *P. vivax *transmission.
4.	Reducing *P. vivax *transmission will have a smaller than currently predicted effect on the incidence and prevalence of vivax malaria initially.
5.	Reducing *P.falciparum *transmission may reduce the incidence of *P.vivax *infections and reduce *P.vivax *transmission. However any effect on the incidence of clinical disease would probably be delayed because reducing falciparum malaria will also reveal vivax malaria by lifting suppression in mixed infections, and a reduction in vivax incidence will reduce immunity. A single radical treatment for all malaria infections may be justified in areas where both parasites are prevalent (i.e. ACT + radical primaquine regimen) [143].
6.	It is very difficult to exclude the presence of long-latency *P. vivax *phenotypes in studies conducted in vivax endemic areas. Long-latency phenotypes may be prevalent over a much wider area of the tropics than currently thought.
7.	Assessment of the efficacy of interventions requires characterization of the prevalent relapse phenotypes.
8.	Assessments of radical curative activity where long-latency phenotypes are prevalent require one year's follow-up. Genotyping should assist in assessing long-latency relapse.
9.	The proportion of genotypically different (heterologous) relapses will fall as transmission intensity falls

#### Radical treatment therapeutics

In therapeutic assessments a follow-up period of six months or less may miss a significant proportion of the relapses. If the majority of relapses in endemic areas derive from heterologous latent hypnozoites then malaria control interventions which are effective will not prevent relapses emerging for months or years, although their number will reduce as the reduced transmission will result in less illness and therefore less hypnozoite activation. Mass drug administration with radical curative regimens (currently primaquine is the only option) would be the only way to eliminate this reservoir of infection quickly. Epidemiological assessments in older children and adults in endemic areas may underestimate the burden of vivax malaria as partial immunity (and premunition) will ameliorate disease severity and may lead to reduced activation of relapses. This would result in relatively low relapse rates. The proportion of acute falciparum malaria infections, which are followed by *P. vivax *may be a better indicator of the prevalence of latent hypnozoite carriage.

It is possible that much of the variance in responses to primaquine is explained by differences in rates and burdens of latent hypnozoite carriage and degree of immunity and not variation in drug susceptibility. The same factors which affect therapeutic responses in the blood stage infection appear to affect the responses to hypnozoitocidal treatment i.e. organism load and immunity. We have tended to consider 8-aminoquinoline efficacy only from the perspective of the drug, and we do not take into account either organism load, organism phenotype, or host immunity. Taking a quantitative approach to assessing 8-aminoquinoline radical curative activity based on hypnozoite burdens may be a valuable approach. Patients with very large liver burdens of hypnozoites from either a very heavy inoculation or multiple inoculations and little or no immunity (such as soldiers) would be expected to have a larger number of relapses than travellers who have a brief period of exposure. Studies of radical curative activity in soldiers may not be comparable to studies in travelers. Studies in adults may not be comparable to studies in children. The apparent radical curative activity of primaquine would be expected to improve as malaria transmission falls. Lower dose regimens may have useful efficacy in this context. Long term follow up (minimum one year) of well characterized patients with parasite genotyping in low transmission settings should help to dissect the contributions of pre-existing versus recently inoculated hypnozoites to relapse. These should be accompanied by entomology and genotyping studies in wild-trapped anopheline vectors to determine mixed genotype and genetic recombination rates.

#### Immunity

It is interesting to speculate on the role of relapse in enhancing the opportunities for recombination and how, in the pre-anti-malarial era, relapse would have contributed to persistence of the untreated infection. Rechallenge experiments showed that a "strain specific" immunity developed after protracted symptomatic infection with that *P. vivax *strain. But this did not always prevent subsequent parasitaemia, particularly if rechallenge was many years later [163]. Despite considerable investment in a *P. vivax *vaccine there is relatively little information how immunity to vivax malaria is acquired and maintained in the context of frequent infection and relapse with different genotypes [164]. It is generally recognized that asymptomatic *P. vivax *infections are common in endemic areas but their overall contribution to *P. vivax *transmission in malaria-endemic areas, and the importance of relapse in maintaining these asymptomatic infections has not been characterized adequately. However the malaria therapy experience suggested that asymptomatic infections were a very important source of infection [163]. An effective vaccine which did not confer long-lasting immunity and required frequent boosting might provide a selective pressure towards longer latency.

There are major questions about the basic biology and the epidemiology of *Plasmodium vivax *relapse which first need recognizing, and then need answering, if we are to address seriously controlling and eliminating this important malaria parasite [165].

## Competing interests

The author declares that they have no competing interests.

## Authors' contributions

I wrote, read, and approved the final manuscript.
